# *Candida albicans* releases a peptide from the Rbt1 protein to promote its invasion into the gut epithelium

**DOI:** 10.1080/19490976.2025.2573038

**Published:** 2025-10-30

**Authors:** Hervé Bègue, Amandine Ducreux, Tracy Paradis, Alicia Loiselet, Thierry Mourer, Géraldine Lucchi, Sylvie Kieffer-Jaquinod, Yohann Coute, Bernhard Hube, Ilias Theodorou, Sophie Thenet, Benjamin Gillet, Sandrine Hughes, Pierre Lapaquette, Louise Basmaciyan, Sophie Bachellier-Bassi, Christophe d'Enfert, Fabienne Bon, Frédéric Dalle

**Affiliations:** a UMR PAM Univ Bourgogne Europe - Institut Agro Dijon - INRAE, Equipe Aliment, Fermentation, Interaction, Microbiote - AFIM, UFR Sciences de Santé, Dijon, France; b Proteomic Plateforme Clips, Dijon, France; c Institut Pasteur, Université Paris Cité, INRAE USC2019, Unité Biologie et Pathogénicité Fongiques, Paris, France; d Univ. Grenoble Alpes, INSERM, CEA, UA13 BGE, CNRS, CEA, Grenoble, France; e Department of Microbial Pathogenicity Mechanisms, Leibniz Institute for Natural Product Research and Infection Biology-Hans Knoell Institute (HKI), Jena, Germany; f Institute of Microbiology, Faculty of Biological Sciences, Friedrich Schiller University, Jena, Germany; g Sorbonne Université́, INSERM, Centre de Recherche Saint-Antoine, Paris, France; h EPHE PSL University, Paris, France; i Plateforme de Séquençage de l'IGFL, Lyon, France

**Keywords:** Rbt1, *Candida albicans*, enterocytes, tight junctions

## Abstract

*Candida albicans* has been recently added to the WHO critical priority group of fungal pathogens based on its impact on global public health. *C. albicans* is a mucosal commensal yeast in humans that can cause severe gastrointestinal-borne disseminated candidiasis in immunocompromised patients. *C. albicans* interaction with enterocytes is thus a key step in the pathophysiology of disseminated candidiasis. Here, we show that, during the first steps of the infection process, *C. albicans* releases a peptide from the Repressed by Tup1 protein 1 (Rbt1), which disorganizes the cell‒cell adhesion junctions in Caco-2 intestinal epithelial cells by notably downregulating constitutive proteins of the tight junction complex (i.e. ZO-1). This is the first report to show that a peptide of a human pathogenic fungus promotes fungal invasion into the gut epithelium by disorganizing the protective epithelial barrier.

## Introduction

*Candida albicans* is a fungal pathobiont residing as a commensal on the surface of mucosa in healthy humans as the result of a balanced interplay between the fungus, the microbiome, and host defenses. Disruption of this balance triggers the expression of fungal traits associated with pathogenicity.^1-7^ Consequently, *C. albicans* behaves as an opportunistic pathogen that can be responsible for infections ranging from superficial to disseminated candidiasis in vulnerable individuals. Disseminated candidiasis is mainly due to excessive colonization of the gut mucosa followed by the translocation of the fungus through the gut barrier in immunocompromised patients.^5^^,^^8^^,^^9^ Therefore, the interaction between *C. albicans* and intestinal epithelial cells IEC is crucial either during the establishment of commensalism or during the successive steps of fungal infection.^10^^,^^11^

To translocate through epithelial layers and damage epithelial cells, *C. albicans* has developed several strategies whereby the yeast-to-hyphae transition plays a pivotal role.^12^^,^^13^ Whereas both trans- and para-cellular translocation routes have been reported,^12^^,^^14^ transcellular crossing remains the main mechanism associated with epithelial cell damage. This involves two mechanistically distinct routes of invasion: (i) epithelial-driven endocytosis of *C. albicans* hyphal tips, involving the interaction of fungal hyphal-associated surface invasins with defined membrane epithelial receptors (including E-cadherin, EGFR/HER2 and c-Met),^15^ and (ii) the active penetration of fungal hyphae is potentiated by the release of fungal components. Whereas epithelial endocytosis initiates and potentiates the invasion process of *C. albicans* hyphae into epithelial cells, active penetration of hyphae exerts a pressure upon infected epithelial cell surfaces that strongly deforms epithelial plasma membranes, creating a pocket of epithelial invasion favoring the accumulation of fungal molecules secreted extracellularly by *C. albicans* hyphae. In fine, epithelial cell damage is attributed to the mechanical deformation of plasma membranes driven by hyphal growth in association with the cytotoxicity induced by secreted fungal molecules. These molecules include secreted hydrolases and the pore-forming toxin candidalysin, whose functions have been well described as inducing alterations in cellular junctions and the plasma membrane, tissue damage, inflammasome activation, and finally cell death.^10^^,^^11^^,^^15-21^

Whereas both invasion routes require initial adhesion of the fungus to epithelial cells,^11^ cellular endocytosis of *C. albicans* was initially described as a cell type-dependent mechanism observed in both vaginal and oral-epithelial cells.^17^^,^^22^ In contrast, in IEC the tight junction (TJ) complex limits fungal invasion by masking epithelial receptors involved in the induction of the endocytic pathway.^23^ Interestingly, we have previously shown that pharmacologically altering the TJ complex facilitates *C. albicans* invasion into IEC by allowing interaction of fungal invasins with epithelial cell receptors triggering the endocytic machinery.^23^

Here, we report early gut barrier breakdown by *C. albicans* cells interacting with polarized IECs, namely, Caco-2 cells, correlated with the downregulation of cell–cell adhesion proteins, including different constitutive proteins of the TJ complex. While this pathogenic process does not require *C. albicans*-IEC contact, it does involve *C. albicans*-secreted molecules, including a peptide released from the Repressed by Tup 1 (Rbt1) protein. Whereas this fungal GPI-anchored cell wall protein was previously reported to be involved in mating efficiency, biofilm formation, adhesion to cell surface proteins of the host and in cell‒cell aggregation,^24-26^ we report herein the discovery of a novel function of Rbt1 as a virulence factor weakening the barrier function of the gut layer. This mechanism secondarily favors *C. albicans* invasion into IECs during the early stages of the infection process.

## Materials and methods

### Intestine epithelial cell (IEC) line and growth conditions

The Caco-2 intestinal epithelial cell line derived from a human colon adenocarcinoma (ATCC n°HTB-37™) was grown in Dulbecco's modified Eagle's medium (Glutamax and high glucose, 4.5 g L^−1^) (DMEM) (Gibco, Life Technologies) supplemented with 10% (v/v) FBS (PAN Biotech) and 0.1 mM non-essential amino acid mixture (Dominique Dutscher). For permeability assays (TEER and LY) and microscopic observation of TJ proteins after immunofluorescence staining, cells were seeded on Transwell® inserts (polyethylene terephthalate membrane, pore size = 0.4 μm, effective growth area of the membrane = 0.3 cm2) (Falcon, Corning) at a rate of 1.25 × 10^5^ cells per well. Evaluation of Caco-2 cell monolayer grown on permeable supports complied microscopic observation of the aspect of the cell monolayer and routine TEER measurements. If the TEER value of a well was less than 1400 *Ω*.cm^2^, this well was rejected. For the WB and invasion assays, 2.5 × 10^5^ cells were seeded onto 14 mm diameter glass coverslips in 24-well plates. The cells were maintained in a humidified incubator at 37 °C with 5% CO_2_, and the medium was changed every 2−3 d. The cells were passed every 7 d in flasks. They were used for experiments 15 to 21 d after seeding (passages 10–20).

### *C. albicans* strains and growth media

*C. albicans* SC5314 (*Ca*-SC5314) was used for all the assays as a wild-type strain.^27^ To clarify the role of *C. albicans* on tight junction alteration, different mutants were used as specified in Table 1. All strains were maintained on solid Sabouraud dextrose agar 2% (m/v) with gentamicin and chloramphenicol (BD). The *rbt1∆KR* mutant was constructed in the *rbt1* KO mutant as follows: the 3ʹUTR region of *RBT1* was amplified on *Ca*-SC5314 genomic DNA using primers ThM85 and ThM86 (Table 2) with high-fidelity polymerase Q5 according to the manufacturer's instructions; the fragment was then digested with SacI and SacII and inserted into pSFS2A cut with the same enzymes, yielding pSFS2A-3ʹUTR_*RBT1*_. The *RBT1* coding sequence mutated for the two proximal Kex2 sites was obtained by fusion PCR; two PCR fragments were amplified with Q5 polymerase on *Ca*-SC5314 genomic DNA with the primers ThM82 and ThM12, and ThM62 and ThM81, respectively, introducing deletions of K59, R60, K73, and R74. The two fragments were then fused using primers ThM82 and ThM81. The resulting fragment was digested with KpnI and XhoI and inserted into the pSFS2A-3’UTR_*RBT1*_ cut similarly, yielding pSFS2A-*RBT1*^*∆K59,R60,K73,R74*^. The plasmid was digested with KpnI and SacI, and the fragments used to transform *C. albicans* using the lithium acetate-PEG protocol.^28^ The transformants were selected on YPD medium supplemented with 200 µg mL^−1^ nourseothricin after 48 h of incubation at 30 °C. Proper integration at the *RBT1* locus was checked by PCR using the primers ThM3 and ThM4.

**Table 1.** *C. albicans* strains used in this study.

Strain name	Strain/gene function	Relevant genotype	Reference
SC5314	Wild-type		[27]
CAI-4	Parental strain (*rbt1Δ/Δ)*	*ura3∆:λimm* ^*434*^ */ura3∆:λimm* ^*434*^	[29]
CAY171	Parental strain (*rbt1KR)*	*arg4∆/arg 4∆ rbt1∆:LEU2/rbt1∆:HIS1*	[24]
*rbt1Δ/Δ*	Adhesion and biofilm formation	*rbt1∆:hisG/rbt1∆:hisG-URA3-hisG ura3∆:λimm* ^*434*^ */ura3∆:λimm* ^*434*^	[30]
*kex2∆/∆*	Killer expression 2, serin protease	*arg4∆/arg4∆ kex2∆:LEU2/kex2∆:HIS1*	[31]
*rbt1∆KR*	Deletion of -KR- dipeptide flanking the *RBT1* coding region (*rbt1∆KR*^*59-60*^*∆KR*^*73-74*^*)*	*arg4∆/ARG4 rbt1∆:LEU2/rbt1∆:HIS1* P_*RBT1*_*-V5-rbt1*^*∆K59,R60,K73,R74*^*-SAT1*	This study

**Table 2.** Oligonucleotides used in this study.

Name	Sequence
ThM3	CATTTTATCCACTGAGGCTACTTTCC
ThM4	CAGAAGAATCACTCTTGAAGTTACAATC
ThM12	ATCAATACCATAAGAACCGTCAGATAATTTTTTGGCATTAGACACGACAAATGCGGCATTGAAACCGGCATTTACACCA GCGCTTCCAAAAGTTTCAATTGTACCATCTTTATTGGCAAT
ThM62	GACTTGGGTCTTTACCACGATAGCTCCATTTCtCTTGGTGGTTCAAAGAACGGTAAACCAATTCCAAATCCATTGTTGGG TTTGGATTCAACTGAAGCTGAAATTGCCAATAAAGATGGT
ThM81	ATCACTCGAGTTAGATCAAGAATGCAGCAAGACCAATAATAG
ThM82	ATCAGGTACCCATAATGTGATATTTGATAACTCTGGCACAG
ThM85	ATCAGAGCTCTTCTAGTTACTGATACTATATCTTTTTCTTTTTC
ThM86	ATCACCGCGGGTATCCTAACCATTAAATCTTTAATCGCCGAAC

For the experiments, after an overnight growth on YPD broth (MP Biomedicals) at 37 °C, 300 rpm in an Ecotron incubator (Infors HT), *C. albicans* cells were diluted in fresh liquid YPD to an OD_600 nm_ of 0.28 to 0.32 and grown to log phase for another 2 h under the same conditions. The cells were then adjusted to the desired concentration in DMEM without FBS.

For “killing” assays, the yeasts were incubated in phosphate-buffered saline (PBS, 8 mM Na_2_HPO_4_, 2 mM KH_2_PO_4_, 140 mM NaCl, 2.7 mM KCl, pH 7.2) containing 0.04% (m/v) Thimerosal (2-[ethylmercuriomercapto] benzoic acid sodium salt, Sigma) for 1 h at room temperature on a shaking plate. After washing them three times with PBS, they were adjusted to the desired concentration in DMEM. Fungal inhibition was confirmed by plating 100 µL of treated cells on Sabouraud agar.

### Secretome production

Different supernatants of *C. albicans* subcultures were obtained in DMEM w/o FBS at 37 °C in an Ecotron incubator (Infors HT) (300 rpm) either at 10^7^ yeasts mL^−1^ for one to four h or at 5.3 × 10^7^ yeasts mL^−1^ for 1 h. After centrifugation for 10 min at 1065 g, the supernatant was sterilized by filtration. For determination of the size of the molecule of interest, different Amicon Ultra 15 mL centrifugal filters (Merck, Millipore) were used. All the supernatants were tested without any dilution. The samples were directly used or stored at −20 °C for up to one week.

### Physical analysis by ATR-FTIR

Fourier transform infrared (FTIR) spectra were acquired by attenuated total reflection (ATR) using a Thermo Scientific Nicolet iS5. *Candida* supernatant and DMEM medium were gently placed on the surface of the diamond crystal and pressed with a constant force with a torque screwdriver. Each spectrum was recorded from 650 to 4000 cm^−1^ with a 4 cm^−1^ resolution resulting from the accumulation of 32 scans.

### Synthesized peptides

Rbt1 peptides were dissolved in acetonitrile:H_2_O (1:3) as recommended by the manufacturer (Proteogenix) at a stored concentration of 22 mM (Table 3). They were used at concentrations comprised between 0.1 and 100 µM in DMEM, with the absence of cell cytotoxicity being systematically verified (10–100 μM).

**Table 3.** Amino acid sequences of peptides used in this study. Rbt1 protein accession number Q59TP.

Name	Position	Amino acid sequence
sRbt1^61–72^	61−72	EAEIANKDGTIE
sRbt1^227–242^	227−242	ELDEFEELSNDGVTHS
sRbt1^61–72s^	Scrambled peptide of sRbt1^61–72^	DEGIKNETAEAI
sRbt1^227-242s^	Scrambled peptide of sRbt1^227–242^	DGVEHTSELDENSFLE

### Invasion and adherent assays of *C. albicans* in Caco-2 cells

Invasion and adherence assays of *C. albicans* on Caco-2 cells were performed as previously described.^17^ Briefly, intestinal cells cultured on coverslips or inserts were infected at MOI 0.1 for 30 min (adherence) or 2 h (invasion) at 37 °C with *C. albicans* killed or not. Infected cells were fixed with 4% (v/v) PFA. The adherent part of *C. albicans* was stained with a rabbit polyclonal antibody (Acris Antibodies) and Alexa-Fluor 568 goat anti-rabbit antibody (Life Technology). After permeabilization of the monolayers, both the adherent and internalized fungal parts were stained using the anti-*C. albicans* antibody and then with Alexa-Fluor 488 goat anti-rabbit antibody (Life Technology). Coverslips were observed with a BX51 fluorescence microscope at ×40 magnification (Olympus). The percentage of invading *C. albicans* cells was determined by dividing the number of total or partially internalized cells by the total number of interacting *Candida* cells (i.e. adherent + invading cells). At least 100 fungal cells were counted on each coverslip, and all experiments were performed in duplicate in at least three independent experiments.

The contribution of intestinal cell endocytosis in the internalization of *C. albicans* was investigated using cytochalasin D (CytD), an inhibitor of endocytosis.^17^^,^^32^ Invasion assays were performed as described above, except that the Caco-2 cells were pre-treated for 30 min at 37 °C with 0.5  μM CytD (Sigma) or with an equivalent amount of its solvent (i.e. DMSO) as a control. Caco-2 cells were further incubated with the inhibitor throughout the infection period. Both innocuities of CytD and DMSO on Caco-2 and *Candida* cells were verified elsewhere at the concentrations used in this study.^17^

### Evaluation of tight junction integrity

The integrity of the tight junctions and the permeability of the epithelial monolayer were studied by monitoring the passage of Lucifer Yellow (LY) during infections (in the presence of yeasts) or by measuring the transepithelial electrical resistance (TEER) when Caco-2 cells were treated either with supernatant or with peptides. TEER was measured using a Millicell-ERS volt-ohmmeter (MerkMillipore). Before each experiment, the differentiation of the Caco-2 monolayer was argued by TEER values between 450 and 800 *Ω* cm^2.^^33^^,^^34^ As initial TEER values vary among different Caco-2 cell monolayers, all the TEER values are expressed as percentage relative to the initial value of each culture.

An increase in permeability was confirmed by evaluating the paracellular transport of the fluorescent molecule Lucifer Yellow (LY) (457 Da) (Sigma) across Caco-2 monolayers. IEC cultured on transwell inserts were incubated with non-cytotoxic concentration of LY (1 mg mL^−^^1^), either in DMEM or together with *C. albicans* strains. After 7 h of incubation, the quantity of LY that crossed from the apical to the basal chamber was assessed by measuring the fluorescence using SAFAS, Monaco, and XENIUS XC (*λ*_ex_ = 425 nm, *λ*_em_ = 540 nm). The quantity of LY was determined based on the difference in fluorescence (T_7 h_—T_0 h_). All values are expressed as percentage relative to the control.

### Immunofluorescence staining of junctional proteins of Caco-2 cells and image analyses

After the experiments, Caco-2 monolayers were rinsed twice with cold PBS + (1  mM CaCl_2_, 0.5  mM MgCl_2_ in PBS). They were fixed and permeabilized with methanol for 5 min at -20 °C and then blocked for 2 h with 3% (m/v) BSA in PBS+ . Tight junctions were revealed using the monoclonal mouse (IgG1) anti-ZO1 antibody (clone ZO1-1A12, Thermo Fischer Scientific) at 4 °C overnight, followed with a goat anti-mouse (IgG) antibody conjugated with AlexaFluor 568 (Invitrogen) for 1 h at room temperature. Finally, the cells were examined with a BX51 epifluorescence microscope (Olympus). For each biological replicate (*n* = 3), at least 20 pictures were acquired with a 60× objective lens using Cell F software, each time with the same exposure parameters and a resolution of 4080 × 3072 pixels.

For picture analysis, the automatic spectral stretching was done using the Fiji software to evaluate the regularity/continuity of ZO-1 labeling at cell‒cell contacts. Then, at least 30 segments of membrane were manually defined, with a mean length of 300 pixels. Particular attention was given to selecting segments that were as straight as possible. The sub-images were cropped to isolate the given segment. Finally, the fluorescence signal value was acquired throughout the segment.

### Immunoblotting of tight junction proteins

IEC membrane proteins were extracted with ProteoExtract® Native Membrane Protein Extraction Kit following the manufacturer protocol (Calbiochem). The protein concentration was determined using Bicinchoninic Acid Kit (Sigma-Aldrich). IEC whole-cell proteins were extracted by directly adding 200 µL of 1.25× Laemmli sample buffer (Bio-Rad) to the cell monolayers. The lysates were then disrupted by sonication for 5 min, and the proteins were denatured by heating at 95 °C for 5 min. Protein extracts were clarified by centrifugation for 10 min at 4000 × g at room temperature.

Equal quantity of membrane proteins or equal volume of total lysate were subjected to SDS‒PAGE (4–15% mini-Protean precast protein gel, Bio-Rad) and transferred to nitrocellulose membrane (Trans-blot turbo, Bio-Rad). After blocking for 1 h with 3% bovine serum albumin (Sigma), the membrane was incubated overnight at 4 °C with anti-GAPDH (clone 258, Invitrogen), anti-occludin (clone OC-3F10, Invitrogen®) and anti-ZO-1 (clone ZO1-1A12, Thermo Fischer Scientific) primary antibodies as recommended by the manufacturers. A goat anti-rabbit or anti-mouse IgG (H + L) DyLight™ 680 and 800 Conjugate (Cell Signaling) antibody was used as the secondary antibody. The blots were developed using Odyssey® Fc Imaging System (LI-COR®) and analyzed with Image Studio Lite 5.2. software.

### Statistical analysis

All the experiments were conducted independently at least three times. The bar plot shows the mean of the replicates with standard error (SEM). Details of each analysis are indicated in the figure legends.

## Results

### *C. albicans* infection of IEC is associated with an early increase in paracellular permeability that correlates with the loss of proteins in the TJ complex

For better understanding, a diagram illustrates the study strategy used here (Figure S1). The permeability of IEC monolayers challenged with *C. albicans* was first investigated using trans-epithelial electrical resistance (TEER) as a readout of permeability (Figure S2a–c). Two inocula of *C. albicans* were tested, mimicking the yeast burden during gut colonization (multiplicity of infection (MOI) 0.1) and infection (MOI 10).^16^^,^^35^ An increase in TEER values was observed during the first 6 h of *C. albicans* challenge. This increase was more pronounced at MOI of 10 compared to MOI of 0.1 (Figure S2a). However, it was not specific to *C. albicans* interaction since similar TEER value modulations were observed when challenging IEC with other pathogenic (i.e. *Nakaseomyces glabratus,* Figure S2b) or non-pathogenic (i.e. *Saccharomyces cerevisiae,* Figure S2c) yeasts. Interestingly, other authors observed a similar increase in TEER values in the early steps of infection of the Caco-2/BBe2.1 cell line by *C. albicans* at MOI of 1, classifying this phenomenon as unspecified.^16^ Thus, we hypothesized that the increase in TEER observed in the early stages of infection resulted from the accumulation and/or proliferation of yeasts on the apical side of the monolayer, which artifactually strengthened the fungal/IEC barrier. In the context of fungal infection, TEER measurements were considered inadequate to assess IEC permeability in the early stages of infection by *C. albicans*.

**Figure 1.**
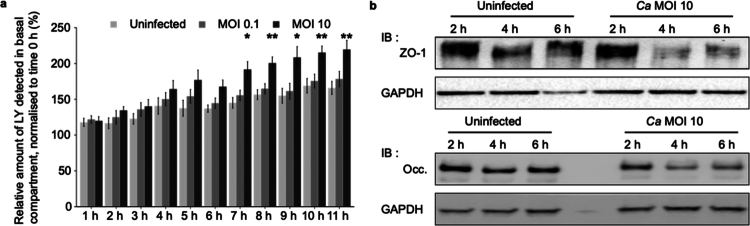
*C. albicans* increases the paracellular permeability of Caco-2 monolayers at the early stage of infection. (a) Caco-2 cells were seeded on Transwell inserts, and experiments were performed after complete differentiation. The monolayer was infected in the apical chamber with two different inocula of *C. albicans* at MOI of 0.1 or 10, and the medium was supplemented with Lucifer Yellow (LY) (1 mg mL^−1^). At each time point, the passage of the LY to the basal chamber was assessed by fluorescence measurement, which was normalized by the basal fluorescence at the onset of infection. Bar plots represent the mean ± SEM of the relative amount of LY detected. For each time point, the value was compared to the uninfected monolayer (Dunn test, *p < 0.05, **p < 0.01, Nb of replicates = 3). (b) Western blot (IB) against ZO-1 and occludin (Occ). A Caco-2 monolayer was infected with *C. albicans* (Ca) for 2, 4, or 6 h. Ten micrograms of membrane proteins and proteins associated with them were used for each experiment. The results depicted here are representative of 3 independent experiments.

To circumvent this technical bias, we assessed the paracellular permeability of IEC by monitoring the passive paracellular transport of the fluorescent small molecule Lucifer Yellow (LY) through IEC monolayers challenged with *C. albicans* at MOI of 0.1 and 10. The accumulation of LY was detectable under all the conditions tested from 1 h to 3 h post-infection (p.i.), as the result of a basal passive transmigration of small molecules through IEC layers, as already reported.^36^ However, an increase in LY accumulation was observed in the basal compartment of IEC monolayers infected with *C. albicans* at MOI of 10 from 4 h *p*.i., which was significant from 7 h *p*.i. (Figure 1a). This observation was consistent with barrier breakdown occurring in the 0–7 h time-period of *C. albicans* infection at MOI of 10, which was exclusively due to an increase in paracellular permeability, since no IEC cytotoxicity was observed during this time-period (Figure S3). Moreover, this result was specific to the *C. albicans* species (Figure S2d).

To specify the kinetics of gut barrier breakdown induced by *C. albicans* infection at MOI of 10, quantification of ZO-1 and occludin proteins was used as a marker of TJ integrity by western blotting IEC protein extracts at 2, 4, and 6 h *p*.i. (Figures 1b, S4a and S4b). As the paracellular transport of LY mainly involves membrane protein complexes,^36^ the protein-extraction protocol was selected to enrich membrane proteins as well as proteins associated with membranes. We showed a decrease in both ZO-1 and occludin markers from 4 h p.i. in IEC infected with *C. albicans* at MOI of 10 compared to control cells (Figures 1b, S4a and S4b).

Collectively, these data showed that *C. albicans* loads, corresponding to infection, increase IEC paracellular permeability in the early stages of infection. This phenomenon is correlated with the modulation of proteins in the TJ complex.

### *C. albicans-*secreted peptides are sufficient to induce barrier breakdown associated with a loss of tight junction proteins.

**Figure 2.**
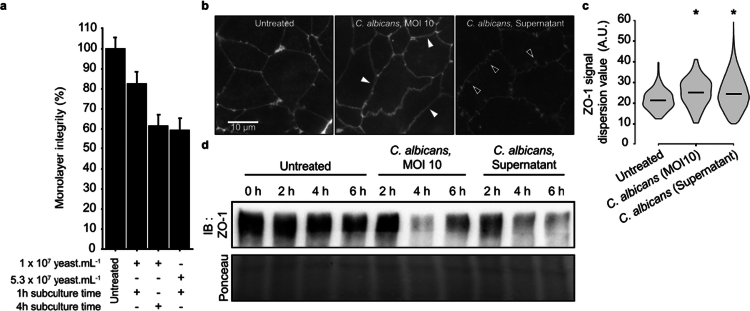
*C. albicans* supernatant is able to modulate the Caco-2 monolayer integrity. Caco-2 cells were seeded on Transwell inserts, and experiments were performed after complete differentiation. (a) Cells were treated with *C. albicans* supernatants in the apical chamber for 1 h 30. Conditions of supernatant production are specified beneath the graphic, in terms of yeast culture duration and/or starting inoculum. The monolayer integrity was assessed using TEER measurement just after adding the supernatant and at the end of the incubation period. The results are displayed as the mean ± SEM percentage of TEER variation compared to the untreated condition (*n* = 3). (b) Immunolabelling of the ZO-1 protein on the Caco-2 monolayer after 1 h 30 min of infection with *C. albicans* at MOI of 10 or treatment with *C. albicans* supernatant (10^7^ yeasts mL^−1^, 4 h-culture). The white arrows highlight cell‒cell separation or thickening of ZO-1 signal, while dark arrows highlight zigzag structures. (c) For immunolabelling experiment, image analysis was performed as explained in Figure S5. Violin plots displaying differences in ZO-1 signal dispersion (A.U.). The higher the value is, the wider is the ZO-1 signal at the cell‒cell contact zone. For this representation, more than 35 000 signals were analyzed. The horizontal line represents the median value. Statistical differences were examined by one-tailed *T*-test; H_0_: mean of dispersion ≤ mean of dispersion for the untreated condition (*p < 0.01). (d) Western blot against ZO-1. Caco-2 monolayer was infected with *C. albicans* at MOI of 10 or treated with *C. albicans* supernatant (10^7^ yeasts mL^−1^, 4 h production) for 2, 4, or 6 h. The whole proteins were extracted, and 5 µL was deposited. The results depicted here are representative of 3 independent experiments.

*C. albicans* interacts with host cells through physical contact and/or via the secretion of molecules (e.g. peptides and/or proteins), or extracellular vesicles EV containing macromolecules such as functional proteasome complexes.^37-39^ Aimed at specifying whether the early increase in permeability was mediated by enterocyte–fungal cell contact or by fungal secreted molecules, we assessed the ability of *C. albicans* culture supernatants to modulate IEC permeability by measuring the TEER variation of IEC monolayers (Figure 2a). The treatment of IEC for 1 h 30 min with sterile *C. albicans* supernatants from a 4 h-culture of 10^7^ *C. albicans* mL^−1^ (i.e. corresponding to MOI of 10), significantly increased IEC monolayer permeability. The treatment was 50% less efficient when using supernatants from a 1 h culture of 10^7^ *C. albicans* mL^−1^.

These observations strongly suggest that secreted molecules were sufficient to alter the permeability of IEC. However, the fungal form that is able to release effective molecules remains to be specified since supernatants were obtained from cultures of mixed fungal forms (i.e. yeast, pseudohyphal, and hyphal forms, in respective proportion of 11.0 ± 1.3%, 35.3 ± 4.2%, and 53.7 ± 3.9%) (Figure 2a). We consequently optimized supernatant production and treated IEC with supernatants of a 1 h culture from a higher starting inoculum of 5.3 × 10^7^ *C. albicans* mL^−1^, which displayed only yeast forms. Interestingly, the supernatants obtained under these conditions showed similar efficiency to the one observed with a 4 h-culture of 10^7^ *C. albicans* mL^−1^ (Figure 2a). These observations provided evidence that *C. albicans* yeast forms are sufficient for the release of active molecules able to alter the TJ complex. Supernatants were obtained from a 1 h culture of 5.3 × 10^7^ *C. albicans* mL^−1^ were used for further experiments and named *Ca*-secretome.

To specify the molecular mechanisms involved in intestinal barrier breakdown, the ZO-1 protein level and spatial distribution were used as markers of TJ integrity^40^ (Figures 2b–d,S4). For both conditions, i.e. *C. albicans* IEC infection and IEC treatment with the *Ca-*secretome, ZO-1 protein levels dramatically decreased in the total IEC protein lysate after 2 h of contact compared to the control condition (Figures 2d and S4c). Importantly, this was not correlated to a decrease in the level of *ZO-1* gene expression (Figure S5). In parallel, microscopic observations revealed tortuous and irregular patterns of ZO-1 immunolabelling (Figure 2b). However, the alteration in ZO-1 distribution differed depending on the treatment conditions. The fluorescence distribution presented discontinuities after 2 h of *C. albicans* infection at MOI of 10, highlighting a gap between the cells (Figure 2b, central panel). In contrast, diffuse signals or zigzag patterns were mainly observed after 2 h of treatment with the *Ca-*secretome (Figure 2b, right panel). All in all, both TJ discontinuities and TJ ruffling, illustrated by zig-zag junctions in IEC were correlated to an increase in the permeability of epithelial layers, as previously reported.^41^ Then, the ZO-1 distribution was specified (Figures 2c, S6) by quantifying the local fluorescence distribution at the cell‒cell contact zone of the IEC. For both infection and treatment with *Ca*-secretome conditions, image analysis of the membrane segments confirmed the wider distribution of the ZO-1 signal at the cell‒cell contact area compared to the untreated condition, for which ZO-1 was focused on a narrower area (Figure 2c).

**Figure 3.**
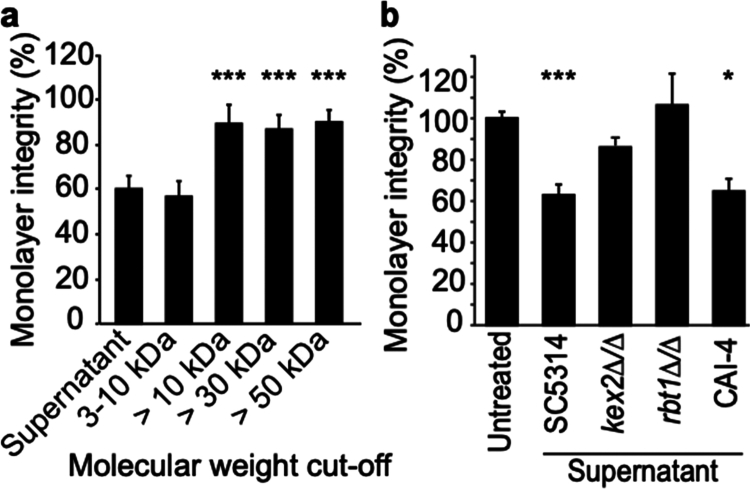
Peptides from the *C. albicans* supernatant are involved in the modulation of monolayer integrity. (a) Identification of the molecular weight range of the molecule of interest. Molecules from the *C. albicans* supernatant (10^7^ yeasts mL^−1^, 4 h production) were concentrated by filtration in different fractions based on molecular weight (3−10 kDa, > 10 kDa, > 30 kDa, or > 50 kDa). The ability of each fraction to modulate tissue permeability was evaluated in differentiated Caco-2 seeded on Transwell inserts. The cells were treated with filtered supernatants at the apical chamber for 1 h 30. The results are displayed as the mean ± SEM percentage of variation compared to the untreated condition, and statistical analyses were performed to compare the effect of the treatment with the supernatant fractions to the complete supernatant (Dunn test, ***p < 0.001, *n* = 3). (b) Identification of the putative proteins of interest as modulator of monolayer integrity through TEER measurement. Supernatants from mutants or from the parental CAI-4 and SC5314 strains were produced (10^7^ yeasts mL^−1^, 4 h-culture), tested on differentiated Caco-2 monolayers, and seeded on Transwell inserts. The cells were treated with supernatants at the apical chamber for 1 h 30 min. The results are displayed as the mean ± SEM percentage of variation compared to the untreated condition, and statistical analysis was performed comparing supernatants to the control condition (Dunn test, *p < 0.05, ***p < 0.001, *n* = 3).

Furthermore, to specify the biochemical nature of *C. albicans* molecules involved in TJ alteration, the Fourier transform infrared (FTIR) spectra of the supernatants were compared to the medium used for its culture (Figure S7b). The supernatants presented lower absorbances within wavelength ranges corresponding to polysaccharides (950−1200 cm^−1^) and lipids (2750−3000 cm^−1^), potentially correlated with their consumption by growing fungal cells. In contrast, a higher absorbance was detected within the wavelength range corresponding to amide bonds and, by extension to proteins and peptides (1200−1750 cm^−1^). This also correlated to an increase in the absorbance at 280 nm, corresponding to the wavelength of aromatic amino acids (Figure S7a). In addition, the supernatant fractions from 3 to 10 kDa induced a similar increase in barrier permeability compared to IEC treated with the complete *Ca*-secretome, which was not observed when testing protein fractions above 10  kDa (Figure 3a).

Finally, given the importance of the Kex2 endopeptidase machinery for the secretion of peptides crucial for *C. albicans* pathobiology,^31^^,^^42-44^ supernatants obtained from a *C. albicans kex2∆/∆* mutant strain (i.e. unable to perform KR-processing of *C. albicans* peptides) were compared to the supernatants of parental strains (Figure 3b). Notably, the supernatants of *C. albicans kex2∆/∆* mutant strain were not able to alter gut barrier integrity, indicating the crucial involvement of the Kex2 machinery in generating peptides able to alter TJ function.

Collectively, these results indicate that, at early stages of infection, Kex2-processed peptides are secreted by *C. albicans* are sufficient to alter IEC barrier integrity by targeting tight junctions.

### The *C. albicans* GPI-anchored protein Rbt1 is involved in tight junction alteration.

We then conducted a peptidomic analysis of the *Ca*-secretome aiming at identifying candidate peptides. Three biological replicates of the 3−10 kDa fraction of the *Ca*-secretome were analyzed by MS-based proteomics. We identified 52 peptides belonging to 35 different proteins in all the biological replicates (Table S1). GO term analysis of the identified proteins revealed that they were mainly associated with the membrane, protein-containing complexes, or extracellular region (Figure S8a).

Based on a strategy similar to that previously used by Le Marquer et al., ^45^ an *in silico* analysis of these 35 proteins identified 169 possible peptides processed by the Kex2 endopeptidase. (Figure S8d). Among them, only two matched the actual identified peptides in the *Ca*-secretome (Figure S8e). Interestingly, both peptides belong to the same protein, Rbt1 (Repressed by Tup1), a member of the hyphal wall protein family. Thus, peptides from Rbt1 appeared to be the only candidates responsible for TJ alteration.

**Figure 4.**
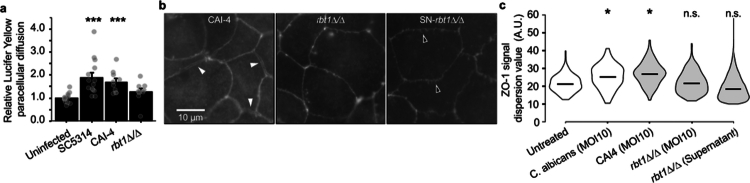
The *C. albicans* GPI-anchored protein Rbt1 is involved in tight junction alteration. (a) Incapacity of the *rbt1∆/∆* mutant strain to increase paracellular permeability at an early stage of infection. The paracellular permeability was assessed on differentiated Caco-2 monolayers seeded on Transwell inserts. The cells were infected with yeast at MOI of 10 in the apical chamber supplemented with LY (1 mg mL^−1^). After 7 h of infection, the LY concentration in the basal chamber was determined. The results are displayed as the mean ± SEM percentage of variation compared to the uninfected condition, and each dot represents the value of a single experiment (Dunn test, ***p < 0.001, *n* > 10). (b) Immunolabelling of the ZO-1 protein in the Caco-2 monolayer after 1 h 30 infection with the *rbt1∆/∆* mutant strain or CAI-4 parental strain at MOI of 10 or treatment with *rbt1∆/∆* mutant supernatant (10^7^ yeasts mL^−1^, 4 h-culture). The white arrows highlight cell‒cell separation or thickening of ZO-1 signal, while dark arrows highlight spike structures. (c) For the immunolabelling experiment, image analysis was performed as explained in Figure S6. Violin plots display differences in ZO-1 signal dispersion (A.U.). The uncolored violin plot indicates the values displayed in Figure 2c. The higher the value is, the wider is the ZO-1 signal at the cell‒cell contact zone. For this representation, more than 35 000 signals were analyzed. The horizontal line represents the median value. Statistical differences were examined by one-tailed *T*-test; H_0_: mean dispersion ≤ mean dispersion for the untreated condition (*p < 0.01). (b–d, g) The monolayer integrity was assessed using TEER measurement just after adding the treatment and at the end of the incubation period.

The involvement of Rbt1 in altering the permeability of IEC monolayers was first confirmed by showing the absence of paracellular transmigration of LY through IEC infected with the *rbt1Δ/Δ* mutant strain compared to the *Ca*-SC5314 and CAI-4 control strains (Figure 4a). This difference was not due to an altered fitness of the *C. albicans rbt1Δ/Δ* mutant strain since all the tested strains had similar growth characteristics (Figure S9). In addition, *rbt1Δ/Δ*-supernatants were not able to increase IEC permeability (TEER measurement) compared to supernatants of both the control strains *Ca-*SC5314 (parental wild-type strain) and CAI-4 (parental strain of the *rbt1Δ/Δ* mutant) (Figure 3b).

The role of Rbt1 was further confirmed by qualitative microscopic observations and quantitative measurements of the distribution of the ZO-1 protein, as previously described in Figure 2b and c, in IEC monolayers infected with *C. albicans rbt1∆/∆* mutant strain or treated with *C. albicans rbt1∆/∆*-supernatants compared to control strains (Figure 4b and c). Similar to *Ca*-SC5314 (Figure 2b, central panel), infection of IEC monolayers with the CAI-4 parental strain showed significant alterations in ZO-1 distribution, with many areas displaying signal that was split on each side of the cell‒cell contact area (Figure 4b, left panel). In contrast, neither infection with the *C. albicans rbt1∆/∆* mutant, nor the treatment with *rbt1∆/∆*-supernatant triggered a disturbance of ZO-1 distribution (Figure 4b, central and right panels). Surprisingly, when treating IEC with *rbt1∆/∆* mutant supernatant, specific structures described as spike structures^41^ were observed (Figure 4b, right panel). However, these structures of unspecified origin were previously reported to be not associated with an increase in the permeability of IEC.^41^ This is in line with our observations showing the absence of modulation of permeability when treating IEC with *rbt1∆/∆*-supernatants (Figure 3b).

Then, ZO-1 distribution was specified by quantifying the local fluorescence distribution at the cell‒cell contact zone of the IEC (Figure 4c). While the distribution of ZO-1 was similarly modified whatever the infectious control strains (CAI-4 or *Ca*-SC5314, Figure 4c), image analyses confirmed the microscopic observations detailed above, showing that ZO-1 distribution was not modified in IEC infected with *rbt1∆/∆* mutants or treated with *rbt1∆/∆*-supernatant compared to the untreated cells. According to the abundance of ECE1 and SAP9 in the secretome, the *ece1ΔΔ* and *sap9-10ΔΔ* mutants were also tested, showing same results as the secretome of the wild-type strain (Figure S10).

In parallel, WB quantification of ZO-1 in Caco-2 treated with *rbt1∆/∆*-supernatant showed the absence of ZO-1 modulation (Figure S11).

Collectively, these data indicate that *C. albicans rbt1∆/∆* mutant as well as *rbt1∆/∆*-supernatant are defective in modulating the TJ integrity, highlighting the involvement of Rbt1 in the alteration of epithelial barrier permeability.

### Rbt1 favors the invasion of *C. albicans* in IEC by altering tight junctions.

**Figure 5.**
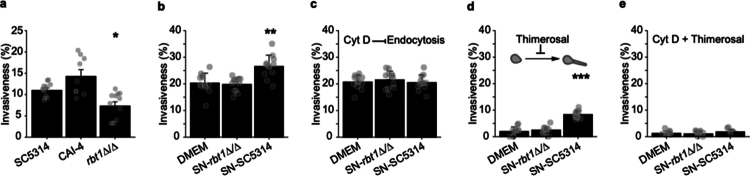
The *C. albicans* supernatant facilitates yeast invasiveness. (a) The invasive capacity of *Ca*-SC5314, the *rbt1∆/∆* mutant strain and its parental CAI-4 strain were measured after 2 h of infection. The results are displayed as the mean ± SEM percentage of invasiveness, and each dot represents the value of a single experiment (100 fungal elements each). Statistical analysis was performed and compared to the *Ca*-SC5314 infection condition (Dunn test, *p < 0.05, *n* > 10). (b, c, d, e) Caco-2 monolayers primed for 1 h 30 min with *Ca*-SC5314 or *rbt1Δ/Δ* supernatants were infected for 2 h with *Ca*-SC5314 at MOI of 0.1. The relative contribution of endocytosis or fungal active penetration were evaluated using an endocytosis inhibitor, cytochalasin D (Cyt D) (0.5 µM), or yeasts inhibited 1 h with 0.04% of Thimerosal. Invasiveness corresponds to the percentage of adherent hyphae partially internalized in primed Caco-2 cells (b). The same experiment was then conducted in the presence of Cyt D (c), during infection with inhibited fungus (d), and during infection with inhibited yeast in the presence of Cyt D (e). The results are displayed as the mean ± SEM percentage of invasiveness, and each dot represents the value of a single experiment. Statistical analyses were performed and compared infection conditions to the control condition (Dunn test, **p < 0.01, ***p < 0.001, *n* > 10).

We observed that the *rbt1∆/∆* mutant displayed less invasiveness than *Ca-*SC5314 (Figure 5a) despite sharing similar adhesion properties (Figure S12), corroborating that Rbt1 is necessary for invasion by *C. albicans*. We thus evaluated the impact of the TJ alteration by *Ca*-secretomes on *C. albicans* invasiveness. For this purpose, IEC monolayers were primed with supernatants obtained from either *Ca-*SC5314 or the *rbt1∆/∆* mutant strain, then the invasiveness of *Ca-*SC5314 was monitored (Figure 5b). Interestingly, the invasiveness of *C. albicans* increased after priming with *Ca-*SC5314 supernatants, which was not observed when priming with *rbt1∆/∆* supernatants. Moreover, the increase in *Ca-*SC5314 invasiveness was abolished when IEC monolayers were primed with *Ca-*SC5314 supernatants in the presence of 0.5  µM of the endocytosis inhibitor cytochalasin D (CytoD) (Figure 5c). These observations confirmed that induced endocytosis contributed to the increase in *C. albicans* invasion at early stages of infection in IEC monolayers with altered TJ^11^^,^^23^^,^^46^ In addition, when treating fungal hyphae with Thimerosal, which prevents active penetration but still allows for induced endocytosis,^17^ a small proportion of thimerosal treated *Ca-*SC5314 cells was still able to be internalized into IEC monolayers primed with supernatants of *Ca-*SC5314. This was not observed in control cells and in IEC monolayers primed with supernatants of the *rbt1∆/∆* mutant strain (Figure 5d). These observations are in line with previous observations reporting that IEC displaying altered TJ are still able to internalize *C. albicans* hyphae defective in active penetration by induced endocytosis.^23^ Finally, when both CytoD and thimerosal were used, no significant *C. albicans* invasiveness was observed despite impaired tissue permeability (Figure 5e).

Collectively, these observations indicate that altering TJ integrity by priming IEC with *Ca*-secretome increases the invasiveness of the fungus by boosting induced endocytosis of *C. albicans*.

### A single Rbt1 peptide is capable of increasing IEC permeability.

**Figure 6.**
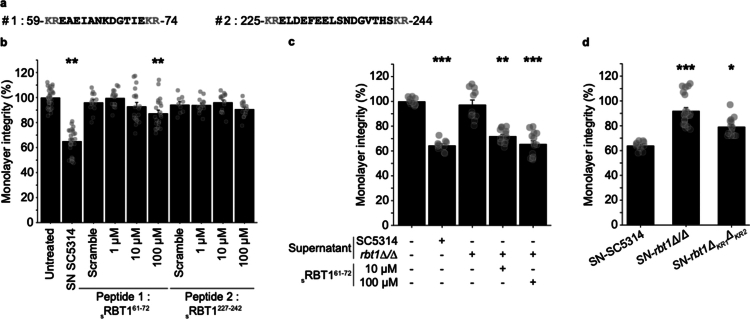
The putative Kex2-generated Rbt1^61–72^ peptide modulates the monolayer integrity but needs the secretome environment to play its full role. (a) Amino acid sequences of the two peptides originating from the Rbt1 protein and identified in MS experiment. Each of them is depicted with the Kex2 machinery cleavage sites (-KR-). (b) Potential modulator role of synthetic peptides sRbt1^61–72^ and sRbt1^227-242^. They were tested on TEER of differentiated Caco-2 monolayers seeded on Transwell inserts. Synthetic peptides ranging from 1 to 100  μM were applied to the apical part of IEC. Their scramble versions were tested at 100  μM. The complete supernatant (SN) was used as a control (10^7^ yeasts mL^−1^, 4 h-culture). (c) The synthetic peptide sRbt1^61–72^ complemented the supernatant of the *rbt1∆/∆* mutant strain to decrease the TEER. (d) The supernatant produced from a *rbt1∆KR* mutant strain was compared to supernatant from the wild-type *Ca*-SC5314 and the *rbt1∆/∆* mutant strain (10^7^ yeasts mL^−1^, 4 h-culture). (b–d) Monolayer integrity was assessed using TEER measurement just after adding the treatment and at the end of the incubation period. The results are displayed as the mean ± SEM percentage of variation compared to the untreated condition, and each dot represents the value of a single experiment. Statistical analyses were performed and compared treatments to the control conditions (b,c) or to the wild-type yeast supernatant (d) (Dunn test,*p < 0.05, **p < 0.01, ***p < 0.001, *n* > 10).

Proteomic analysis identified two Rbt1 peptides from amino acid locations 61−72 and 227−242, respectively, in the 3−10 kDa fraction of the *Ca*-secretome. Both peptides were framed by lysine-arginine (KR) Kex2 processing sites^45^ (Table 3, Figure 6a). The impact of each peptide on barrier integrity (TEER measurement) was tested using synthetic versions (Figure 6b). Only the synthetic peptide_s_ Rbt1^61–72^ induced a significant decrease in IEC permeability at a concentration of 100 µM (Figure 6b). Notably, a reduction in TEER values between 15% and 40% is considered to be of biological importance. This finding is in agreement with previously published works documenting that an alteration of the TJ complex correlated to a reduction in TEER varying between 15% and 50%, depending on the molecules tested (palmitic acid, bile acids, cytokines such as interferon gamma and TNF alpha, lactones).^47-51^ Surprisingly, the increase in permeability observed when treating IEC with the _s_Rbt1^61–72^ peptide was less pronounced than that observed with the *Ca-*secretome. In addition, supplementation of *C. albicans rbt1∆/∆*-supernatants with _s_Rbt1^61–72^ at 10 or 100  µM restored the ability of this supernatant to disturb the IEC barrier (Figure 6c). These findings suggest that the physical environment and/or the presence of other candidate molecules were necessary for the _s_Rbt1^61–72^ peptide to have an optimal biological effect. Finally, the requirement of the Kex2 machinery for the release of the active _s_Rbt1^61–72^ peptide was assessed by testing supernatants obtained from a *rbt1∆KR* mutant strain lacking the KR sites necessary for Kex2-processing of the peptide. As expected, the *rbt1∆KR* mutant produced supernatants that were not able to alter gut barrier permeability (Figure 6d). Taken together, our data reveal that the peptide Rbt1^62–72^ resulting from Kex2-mediated processing of the Rbt1 protein is an important actor of the modulation of IEC permeability.

## Discussion

The consensual model of *C. albicans* translocation through the intestinal barrier is currently described as a stepwise process starting with adhesion and invasion, followed by epithelial damage and loss of epithelial integrity.^12^^,^^14^ Here, we show that, during the early steps of the infection process, *C. albicans* modulates epithelial permeability by targeting TJ integrity, independent of cellular damage following tissue invasion.

Over the years, the secretion of molecules by *C. albicans* has emerged as an important process for pathogenicity.^37-39^ In this study, we report that *C. albicans* supernatants obtained from cultures of wild-type cells increased IEC monolayer permeability. We propose that the alteration of TJ in IEC infected with *C. albicans* occurs through the secretion of active fungal molecules, including secreted peptides. Then, we revealed that the supernatants obtained from the yeast forms had a modulatory effect on TJ strongly suggesting that *C. albicans* hyphae are not essential for the modulation of TJ This is not in line with the observation that the non-filamentous *C. albicans efg1∆/cph1∆* mutant has no impact on IEC permeability.^16^ These apparently divergent observations were reconciled after we discovered the involvement of the Rbt1 protein in TJ alteration since the transcription factor Efg1 is necessary for *RBT1* expression.^30^^,^^52^ The fact that we did not observe any alteration of TJs permeability by using other *C. albicans* mutant strains unable to release pathogenic *C. albica*ns factors (i.e. Ece1 and Sap9-10) strengthen our observations that the phenotype we observed is specific to Rbt1.

However, it was unexpected that two peptides of the Rbt1 protein were identified in supernatants from *C. albicans* yeast cell cultures. In fact, Rbt1 is known to be a member of a hyphal cell wall protein family, and it is reported to be specifically expressed by *C. albicans* hyphae.^24-26^ We provide evidence in this study that the Golgi serine protease Kex2 is involved in Rbt1 processing. Kex2-processing is widely shared in the fungal kingdom, from *Basidiomycotae* to *Ascomycotae*, and from filamentous to non-filamentous members, including *Candida* species and *Saccharomyces cerevisiae*, confirming that the processing by Kex2 is not restricted to a single morphotype.^42^^,^^45^ For instance, the maturation of Ywp1, a yeast wall protein member in *C. albicans*, requires Kex2 processing.^43^ Interestingly, in our experiments, the Ywp1 peptide (–RLMGETPI–), which has been previously identified as part of a Kex2-processed peptide of Ywp1 and proposed as a spacer rather than a bioactive peptide,^43^ was detected in MS analyses of *C. albicans* supernatants. This proves that the Kex2 machinery was active in the experimental setting. We propose that the production of the Rbt1^62-72^ peptide occurs in both morphological stages of *C. albicans*. This is supported by previous observations by Monniot et al. reported the intracellular presence of Rbt1 protein in yeast form in addition to the cell wall of hyphal forms.^25^ Moreover, as the fate of the protein is cell wall localization, Rbt1 is transported through the Golgi apparatus, where it can be processed by machinery.^53-55^

Interestingly, only a single Rbt1 peptide, Rbt1^61–72^, was identified able to modulate IEC monolayer permeability. However, the effect of the peptide was lower when compared to the complete supernatant prepared from cultures of the wild-type *Ca-*SC5314. Finally, when the supernatant of the *rbt1∆/∆* mutant was mixed to the synthetic peptide, alterations of the monolayer permeability were similar to the one observed with supernatants of *Ca-*SC5314 cultures, suggesting that other factors present in the supernatant contribute to the modulatory effect of the Rbt1^61–72^ peptide.

Regarding the potential physiological role of secreted peptides by *C. albicans*, it is important to note that even if the Rbt1 protein is present in most *Candida* species, *in silico* analysis revealed that Kex2 sites allowing the processing of the Rbt1^61–72^ peptide are found mainly in *C. albicans.* Moreover, in some *C. albicans* strains or in very few other *Candida* species that display the homologous region of Rbt1^61–72^, the alanine 66 residue is replaced by a valine residue. Thus, it is plausible that this peptide is specific to *C. albicans,* indicating that this virulence trait is also specific to this fungus and can have evolutionary importance with specific virulence factor. This importance is supported by our observations reporting lower invasiveness of the *C. albicans rbt1∆/∆* mutant strain compared to the wild-type strain, corroborating previous studies using mice models of disseminated candidiasis where the *rbt1∆/∆* mutant strain was defective in pathogenicity.^30^

Finally, the results of this study are in line with our previous observations showing the invasion potentiation of *C. albicans*, notably by triggering the endocytic machinery, when the TJ complex of IEC monolayers is pharmacologically altered.^23^ Here, we show that alterations of the tight junction complex occur, at least in part, through the *C. albicans*-secreted peptide Rbt1^61–72^. Consequently, this alteration facilitates the interaction between hyphal forms of the fungus with the multiprotein complex c-Met – E-cadherin—EGFR/Her2 necessary for triggering induced endocytosis of *C. albicans*, which then potentiates epithelial invasion by *C. albicans.*^15^

## Limitations of the study

Here, we used the Caco-2 cell monolayer to study intestinal epithelial permeability in the context of the interaction of *C. albicans* with the gut barrier. More complex models that were recently developed (e.g. co-culture models, linearized organoids, and microphysiological systems) could be considered more relevant to mimic in vivo-like barrier features,^56^ but they remain difficult to implement and use. In addition, our goals were to specify the molecular mechanisms by which *Candida albicans* deals with enterocytes, independent of the presence of other factors (e.g. the mucus, microbiome, immune cells, etc.) that may interfere with such molecular processes. In this context, the Caco-2 cell monolayer is still considered as a gold standard tool for an enterocyte *in vitro* model for assessing intestinal permeability.^57^ Although it is widely regarded as a good predictive tool for passive paracellular diffusion, several technical caveats must be considered to avoid misleading results. First, passage number and culture duration exert strong effects on barrier properties; therefore, we previously characterized this model in our hands by evaluating culture parameters on cell differentiation in both insert or cover slide models.^23^ This allowed us to drive several previous studies using this model.^17^^,^^23^^,^^58^^,^^59^ Second, considering that cell batch, media and supplementation, substrate/coating materials, and different handling (e.g. TEER measurement protocols) exert strong effects on cellular differentiation and consequently barrier properties, we have improved our protocols by compiling and respecting all the recommendations depicted by both the ATCC and Hubatsch in Nature Protocols.^57^ Additionally, the differentiation of each batch of cells was objectified either by measuring, for each insert, the TEER for tissue permeability to ions^60^ or by arguing the presence of mature TJ by quantifying the ZO-1 protein by WB or microscopic observations in cover slides, as suggested by Hubatsch.^57^ Models that recapitulate the mucus layer or the mechanical forces exerted by flow and peristalsis will be useful to complete our knowledge on Rbt1 effects on the intestinal barrier in future studies.

## Conclusion

We report the discovery of a new strategy developed by *C. albicans* to circumvent the protective role of TJ protein complexes through the secretion of peptides. Moreover, we identified a new virulence function of *C. albicans* Rbt1 protein, which, together with other molecules of the fungal secretome, such as Ece1 and Saps, plays a crucial role in initiating invasion and translocation of the fungal pathogen *C. albicans* through the gut barrier.^24-26^

## Supplementary Material

Supplementary material Figure S1. Diagram illufstrates the strategy used in this study. MOI, multiplicity of infection; TJ, tight junctions; IM, immunomicroscopy; LY, Lucifer yellow permeability experiment; TEER, transepithelial electrical resistance measurement.Figure S2. Permeability of intestinal epithelial cell (IEC) during infection. (a, b, c, d): Permeability of intestinal epithelial cell (IEC) tissue evaluated by transepithelial electrical resistance measurements (TEER) during infection. Differentiated Caco-2 IEC grown in transwell inserts were infected with two different inocula of *C. albicans* SC5314 at MOI 10 and 0.1 (a), *Nakaseomyces glabratus* at MOI 10 and 0.1 (b), or Saccharomyces cerevisiae at MOI 10 and 0.1 (c). The measured values were normalized to the onset of infection and to the untreated condition. The results show the mean and 95% confidence interval. The early phase of infection is highlighted by black dashed lines and gray surface. (d): Permeability of intestinal epithelial cell (IEC) tissue evaluated by LY transport during infection. The monolayer was infected in the apical chamber with *C. albicans*, *Nakaseomyces glabratus* or *Saccharomyces cerevisiae* at MOI 10, and the medium was supplemented with Lucifer yellow (LY) (1 mg mL^−^^1^). After 7 h, the passage of the LY to the basal chamber was assessed by fluorescence measurement, which was normalized by the basal fluorescence at the onset of infection. Bar plots represent the mean ± SEM of the relative amount of LY detected. For each strain, the value was compared to the uninfected monolayer (Dunn test, *p < 0.05, Nb of replicates = 10).Figure S3. Evaluation of Caco-2 cytotoxicity induced by several treatments. After the incubation time necessary to the different conditions, the IEC monolayers were stained with the Sytox nucleic acid strain (Molecular Probes, Life Technology) for 10 min to monitor membrane cytotoxicity (membrane permeation). Immunofluorescence of the monolayers was quantified with a Victor Wallac X4 spectrophotometer (485/535 nm) (PerkinElmer). At least 6 biological replicates were tested, and for each well, 100 measurements were performed on different parts of the IEC monolayer. Methanol was used as a positive control with 100% dead cells. The results are represented as the distribution of Sytox fluorescence intensity, and the threshold indicating a cytotoxicity effect is depicted as a gray dashed line. This threshold corresponded to two-fold of the fluorescence value distribution of the untreated condition.Figure S4. Quantification analysis of ZO-1 and Occludin protein bands density from western blots. A Caco-2 monolayer was infected with C. albicans or incubated with *C. albicans* supernatant for 2, 4, or 6 h. (a, b) Protein extracts were enriched in membrane proteins and proteins associated with them, while (c) protein extracts corresponded to total proteins. For each quantification, the band intensities were corrected by subtracting the background value. Then, the corrected intensity ratio of ZO-1 (a, c) or Occludin (b) bands were calculated by dividing by the GAPDH-corrected intensity bands (a, b) or the Ponceau-corrected intensity bands (c). Finally, each corrected intensity ratio was expressed relatively to the 2-h ratio. Each intensity ratio was statistically compared by *t*-test and the corresponding *p*-value is depicted (b, c *n* = 3, a *n* = 5).Figure S5. Relative accumulation of Occludin and ZO-1 transcripts in Caco-2 cells. The relative amount of transcripts in Caco-2 cells treated with the peptide sRbt1^61–72^ or *C. albicans* supernatant (A) or during yeast infection (B) was quantified by RT-qPCR using THRP1 (A) or GAPDH (B) as a calibrator. Concerning the peptide or supernatant treatment, for each time point, the reference sample chosen was Dulbecco's modified Eagle's medium (DMEM) condition. Concerning the infection condition, for each condition, the reference sample chosen was the starting inoculation time. Data represent the mean value of the fold change ± SD (*n* = 3).Figure S6. Details of the image analysis performed for the ZO-1 immunolabeling experiments. (a) Images depicting different steps of pre-analysis, including denoising of the raw image and selection of the region of interest. Attention was given to select segments of cell‒cell contact zones. (b) Principle of the image analysis of ZO-1 signal. The image is chunked in small parts (red dashed lines on the theoretical images), and the signal is analyzed along a line perpendicular to the cell‒cell contact zone. The shape of the signal (red solid line on the right panel) may correspond to (i) a Gaussian-like signal (right upper panel) corresponding to a non-disrupted ZO-1 signal, (ii) Gaussian curve with shoulder corresponding to a moderately disrupted ZO-1 signal, or (iii) a curve with multiple pics (right bottom panel) corresponding to a disrupted ZO-1 signal. For each signal, the maximum value of intensity is identified and used to normalize the signal (intensity max = 1 and pixel position of the major pic = 0). To determine the ZO-1 signal dispersion value, the width of the curve at 10% of the maximum intensity was determined (green solid line). (c) & (d) example of this strategy on two different images (left panels), corresponding to non-disturbed ZO-1 signal (c) and a disrupted ZO-1 signal (d). For each case, the mean and 95% confidence interval were traced (central panel), and the ZO-1 signal dispersion value through the width of the curve at 10% of the maximum intensity was determined (green solid line, right panel). In those examples, we determined a ZO-1 signal dispersion value of 17 for (c) and a ZO-1 signal dispersion value of 25 for (d).Figure S7. Spectral absorbance of *C. albicans* supernatants. (a) The spectral absorbance of *C. albicans* supernatants obtained after 1 or 4 h of culture were compared to DMEM. The absorbance wavelength was varied from 250 to 600 nm. (b) FTIR spectra (*n* = 3) of DMEM medium and *C. albicans* supernatant (10^7^ yeast mL^−1^, 4 h production). The wavelength range characteristics for carbohydrates, proteins and lipids are highlighted in gray.Figure S8. In silico analysis of *C. albicans* secretome. This analysis compiles the results of three different *C. albicans* secretomes (concentrated 3–10 kDa fraction, see methods SI), and the modulatory effect of these secretomes on the permeability of the IEC monolayer was confirmed. The peptides of interest were those that were identified in all the biological replicates. In total, 52 peptides were selected, corresponding to 35 proteins. GO term analysis was performed on those proteins. The top 10 GO terms are displayed, corresponding to the cellular component (a), the biological process (b), and molecular function (c). In silico cleavage of those proteins was performed following the strategy previously described by Le Marquer et al., ^45^ (d). Venn diagram of putative Kex2-processing peptides and peptides identified within the *Ca*-secretome (e).Figure S9. Growth curves of *C. albicans* strains and mutants. All strains were cultured in liquid DMEM w/o FBS (a) or in YPD (b) at 37 °C with shaking at 300 rpm for 14–16 h. The stationary phase from the pre-cultures was washed 3 times in PBS, and OD was adjusted to 0.1 in culture medium. A volume of 100 µL of cell suspension was placed in a 96-well plate, and growth was monitored by measuring the absorbance at 600 nm every 30 min for 100 cycles at 37 °C in a microplate reader (Plate Reader infinite M200 PRO, Tecan) with orbital shaking (30 s, amplitude: 6 mm, wait: 10 s) before each measurement and multiple reads per well. At least three biological replicates were performed for all the experiments.Figure S10. Identification of the putative protein of interest as a modulator of monolayer integrity through TEER measurement. Supernatants from mutants or parental strains were tested on differentiated Caco-2 monolayers seeded on Transwell inserts. The cells were treated with supernatants from the apical chamber for 1 h 30min . The results are displayed as the mean ± SEM percentage of variation compared to the untreated condition, and statistical analysis was performed comparing supernatants to the control condition (Dunn test, *p < 0.05, ***p < 0.001, *n* = 3).Figure S11. Quantification analysis of ZO-1 protein band density from western blotting. Caco-2 monolayer was treated with rbt1∆/∆ mutant supernatant (SN- rbt1∆/∆) (10^7^ yeasts mL^−1^, 4 h-culture) for 2, 4, or 6 h. The membrane proteins were extracted, and 15 µg was deposited. The results depicted here are representative of 1 experiment.Figure S12. RBT1 gene deletion does not modify the adhesion property of *C. albicans* to intestinal epithelial cells. Differentiated Caco-2 cells were infected with the reference strain SC5314, the rbt1Δ/Δ mutant strain and its parental strain CAI-4 at MOI 0.1. The adhesion of fungal cells to Caco-2 cells was measured after 30 min. The results are represented as bar plots with the mean value of adhesion ± SEM, and the dots represent the adhesion value for each individual experiment. No statistical differences were found (Dunn test, *n* > 10).Table S1. List of peptides identified by MS analysis within the *Ca*-secretome and proteins associated with them. Three different *C. albicans* secretomes (concentrated 3–10 kDa fraction, see methods SI) whose modulating effect on the permeability of the IEC monolayer was confirmed. Peptides of interest are the ones that have been identified in all biological replicates.
