# Supplementary material for: Candida albicans releases a peptide from the Rbt1 protein to promote its invasion into the gut epithelium
Source: Gut Microbes. 2025 Oct 30;17(1):2573038. doi: 10.1080/19490976.2025.2573038 (PMC12578311; doi:10.1080/19490976.2025.2573038)
Supplement: Supplementary material — Figure S1. Diagram illufstrates the strategy used in this study. MOI, multiplicity of infection; TJ, tight junctions; IM, immunomicroscopy; LY, Lucifer yellow permeability experiment; TEER, transepithelial electrical resistance measurement.Figure S2. Permeability of intestinal epithelial cell (IEC) during infection. (a, b, c, d): Permeability of intestinal epithelial cell (IEC) tissue evaluated by transepithelial electrical resistance measurements (TEER) during infection. Differentiated Caco-2 IEC grown in transwell inserts were infected with two different inocula of C. albicans SC5314 at MOI 10 and 0.1 (a), Nakaseomyces glabratus at MOI 10 and 0.1 (b), or Saccharomyces cerevisiae at MOI 10 and 0.1 (c). The measured values were normalized to the onset of infection and to the untreated condition. The results show the mean and 95% confidence interval. The early phase of infection is highlighted by black dashed lines and gray surface. (d): Permeability of intestinal epithelial cell (IEC) tissue evaluated by LY transport during infection. The monolayer was infected in the apical chamber with C. albicans, Nakaseomyces glabratus or Saccharomyces cerevisiae at MOI 10, and the medium was supplemented with Lucifer yellow (LY) (1 mg mL−1). After 7 h, the passage of the LY to the basal chamber was assessed by fluorescence measurement, which was normalized by the basal fluorescence at the onset of infection. Bar plots represent the mean ± SEM of the relative amount of LY detected. For each strain, the value was compared to the uninfected monolayer (Dunn test, *p < 0.05, Nb of replicates = 10).Figure S3. Evaluation of Caco-2 cytotoxicity induced by several treatments. After the incubation time necessary to the different conditions, the IEC monolayers were stained with the Sytox nucleic acid strain (Molecular Probes, Life Technology) for 10 min to monitor membrane cytotoxicity (membrane permeation). Immunofluorescence of the monolayers was quantified with a Victor Wallac X4 spect [file KGMI_A_2573038_SM0459.docx]

**Supplementary Materials for**

**Candida albicans releases a peptide from the Rbt1 protein to promote its invasion into the gut epithelium.**

Hervé Bègue *et al.*

*Corresponding author. Email: frederic.dalle@u-bourgogne.fr

Methods

Mass spectrometry-based peptidomic analysis of *C. albicans* secretome

The 3-10 kDa fraction obtained with Amicon (Merck, Millipore) was acidified using trifluoroacetic acid (TFA). The peptides were then purified using C18 MicrospinColumn (Harvard Apparatus) following the manufacturer’s instructions. After drying down, eluted peptides were solubilized in 5% acetonitrile, 0.1% TFA and analyzed by online nanoliquid chromatography coupled to MS/MS (Ultimate 3000 RSLCnano and Q-Exactive Plus, ThermoFisher Scientific). For this purpose, the peptides were sampled on a precolumn (300 μm x 5 mm PepMap C18, Thermo Scientific) and separated in a 75 μm x 250 mm C18 column (Reprosil-Pur 120 C18-AQ, 1.9 μm, Dr. Maisch). The MS and MS/MS data were acquired using Xcalibur 2.8 (Thermo Fisher Scientific). The mass spectrometry proteomics data have been deposited to the ProteomeXchange Consortium via the PRIDE (PMID: 34723319) partner repository with the dataset identifier PXD053171.

Peptides and proteins were identified by Mascot (version 2.8.0, Matrix Science) through concomitant searches against the Uniprot database (*C. albicans* SC5314 taxonomy, 202402 version) and a homemade database containing the sequences of classical contaminant proteins found in proteomic analyses (keratins…). No enzyme specificity was set, and precursor and fragment mass error tolerances were set at respectively at 10 and 20 ppm. Oxidation of methionine was allowed as variable modification. Proline software (version 2.2.0, PMID: 32096818) was used for the compilation, grouping and filtering of the results (conservation of rank 1 peptides, peptide length ≥ 6 amino acids, false discovery rate of peptide-spectrum-match identifications < 1% PMID: 32970414, and minimum of one specific peptide per identified protein group).

Supplementary Figures

**Fig. S1.**

**
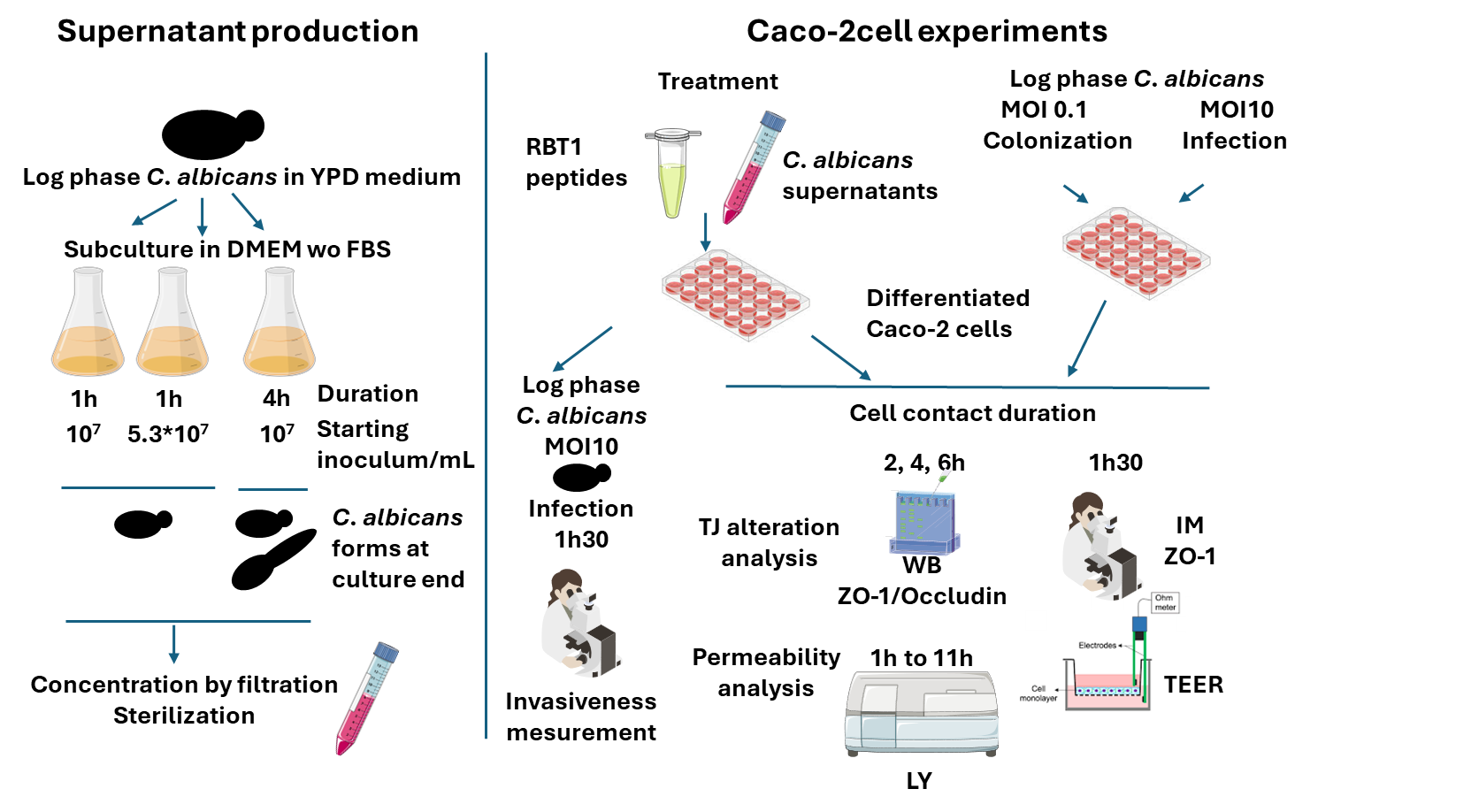
**

**Fig. S1. Diagram illustrates the strategy used in this study.** MOI, Multiplicity of infection; TJ, Tight Junctions; IM, immunomicroscopy, LY, Lucifer Yellow permeability experiment, TEER, Transepithelial electrical resistance measurement.

**Fig. S2.**


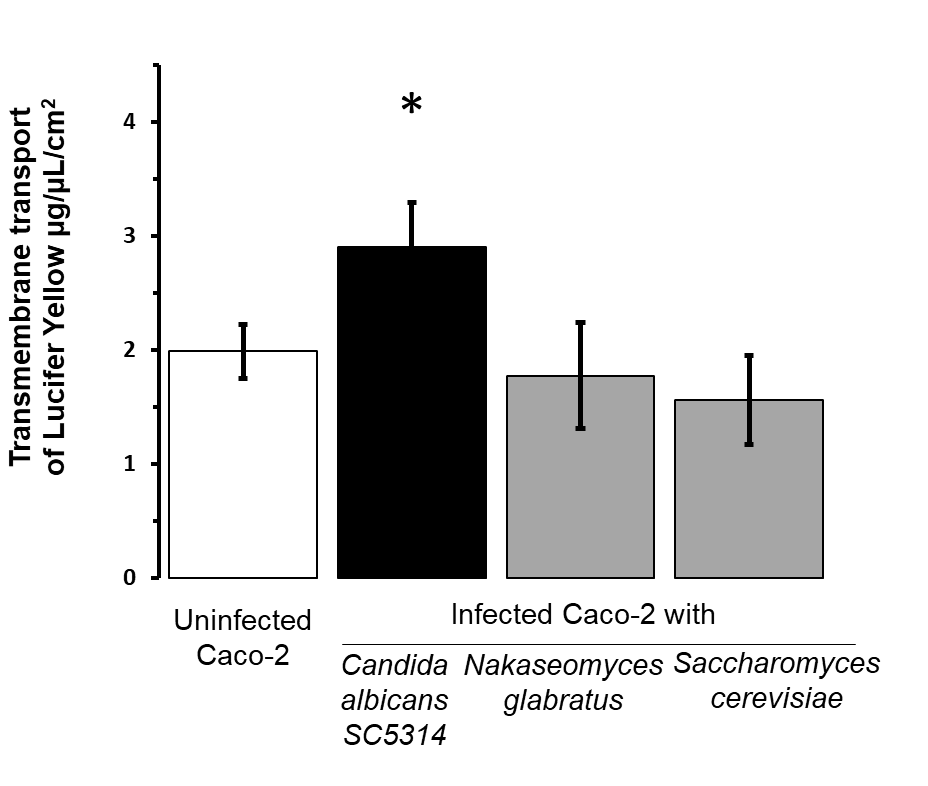


**d**


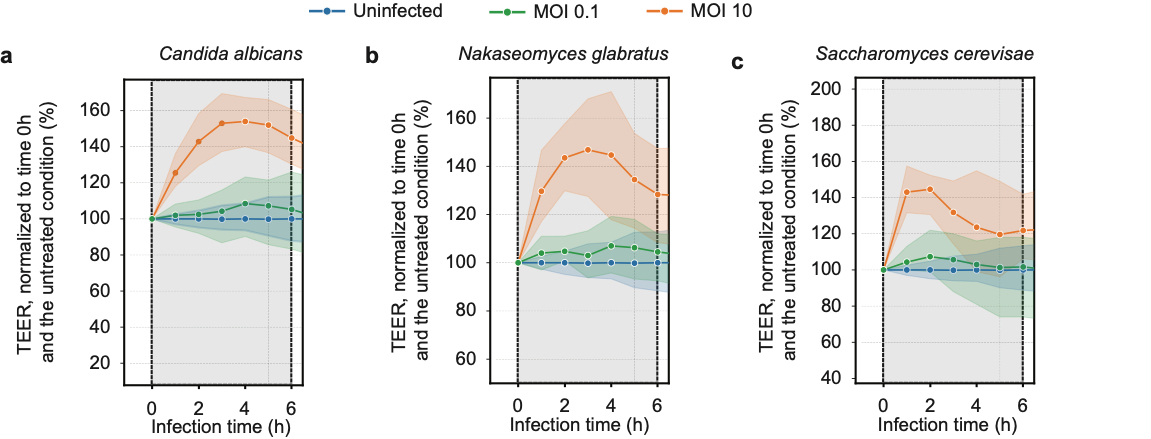
Fig. S2. Permeability of intestinal epithelial cell (IEC) during infection. (a, b, c, d) : Permeability of intestinal epithelial cell (IEC) tissue evaluated by transepithelial electrical resistance measurements (TEER) during infection. Differentiated Caco-2 IEC grown in transwell inserts were infected with two different inocula of *C. albicans* SC5314 at MOI 10 and 0.1 (a), *Nakaseomyces glabratus* at MOI 10 and 0.1 (b), or *Saccharomyces cerevisiae* at MOI 10 and 0.1 (c). Measured values were normalized to the onset of infection and to the untreated condition. Results show the mean and 95% confidence interval. The early phase of infection is highlighted by black dashed lines and grey surface. (d) : Permeability of intestinal epithelial cell (IEC) tissue evaluated by LY transport during infection. The monolayer was infected at the apical chamber with *C. albicans*, *Nakaseomyces glabratus* or *Saccharomyces cerevisiae* at MOI 10, and the medium was supplemented with Lucifer Yellow (LY) (1 mg.mL^-1^). After 7 hours, the passage of the LY to the basal chamber was assessed by fluorescence measurement normalized by the basal fluorescence at the onset of infection. Bar plots represent the mean ± SEM of the relative amount of LY detected. For each **strain**, the value was compared to the uninfected monolayer (Dunn test, * p<0.05, Nb of replicates = **10**).

Fig. S3.


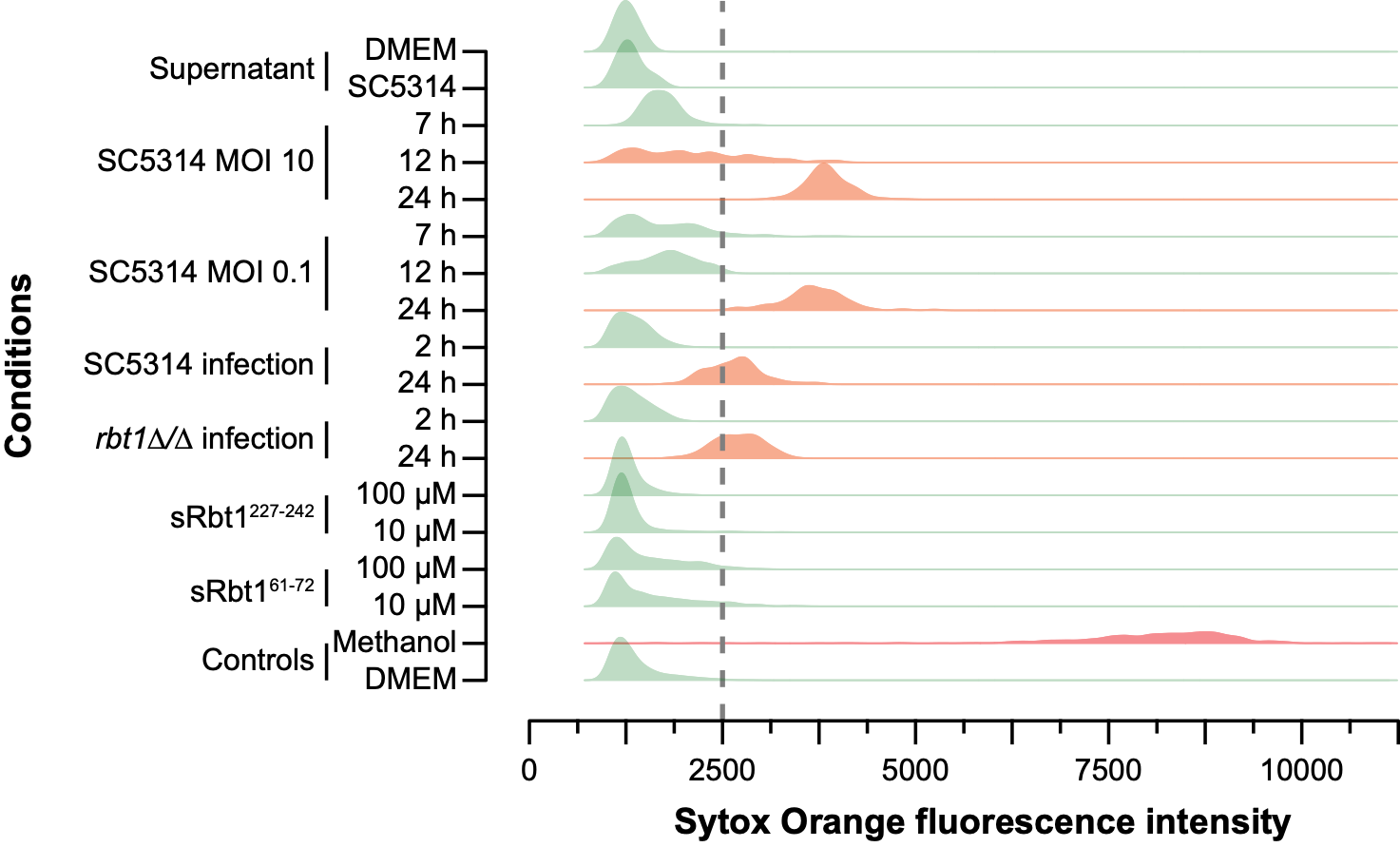
Fig. S3. Evaluation of Caco-2 cytotoxicity induced by several treatments. After the incubation time necessary to the different conditions, the IEC monolayers were stained with Sytox Nucleic Acid Strain (Molecular Probes, Life Technology) for 10 min to monitor the membrane cytotoxicity (membrane permeation). Immunofluorescence of the monolayers was quantified with Victor Wallac X4 spectrophotometer (485/535nm) (PerkinElmer). At least 6 biological replicates were tested, and for each well, 100 measurements were performed on different parts of the IEC monolayer. Methanol was used as positive control with 100% dead cells. The results are represented as distribution of Sytox fluorescence intensity, and the threshold indicating a cytotoxicity effect is depicted as a grey dashed line. This threshold corresponded to two-fold of the fluorescence value distribution of the untreated condition.


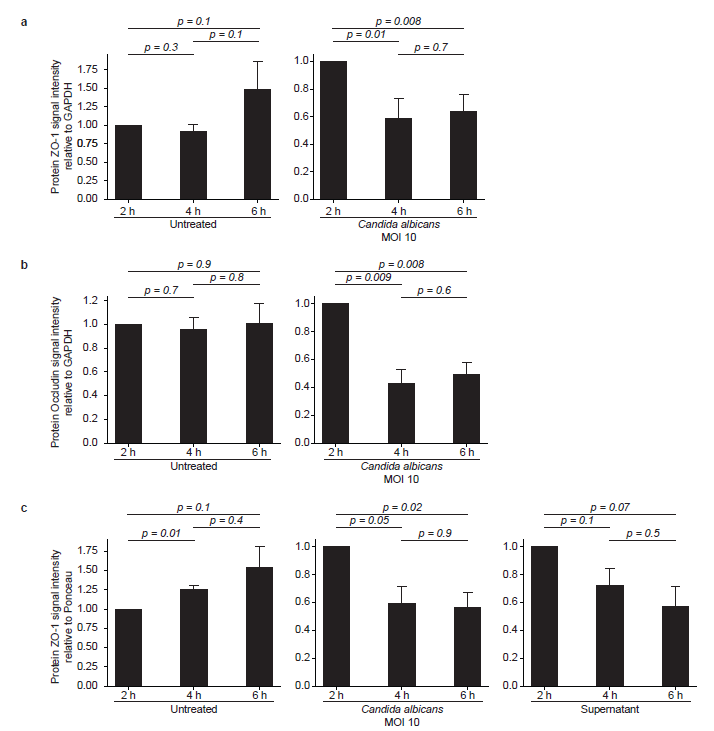
Fig. S4.

Fig. S4. Quantification analysis of ZO-1 and Occludin protein bands density from western blots. A Caco-2 monolayer was infected with *C. albicans*, or incubated with *C.albicans* supernatant for 2, 4 or 6 hours. (a, b) Protein extracts were enriched in membrane proteins and proteins associated with them while

(c) protein extracts corresponded to total proteins. For each quantification, band intensities were corrected by subtracting the background value. Then, the corrected intensity ratio of ZO-1 (a, c) or Occludin (b) bands were calculated by dividing by GAPDH corrected intensity bands (a, b) or the Ponceau corrected intensity bands (c). Finally, each corrected intensity ratio was expressed relatively to the 2 hours ratio. Each intensity ratio was statistically compared by t-test and the corresponding p value was depicted (b, c n = 3, a n = 5).

**Fig. S5.**

**
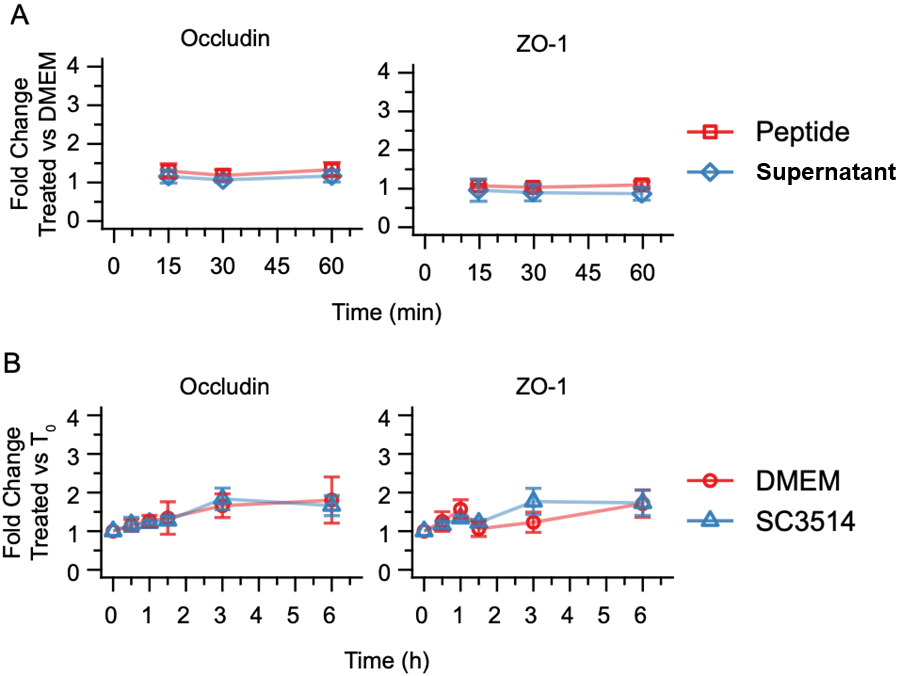
**

**Fig. S5. Relative accumulation of Occludin and ZO-1 transcripts in Caco-2.** The relative amount of transcripts in Caco-2 cells treated with the peptide sRbt1^61-72^ or *C. albicans* supernatant **(A)** or during yeast infection **(B)** was quantified by RT-qPCR using THRP1 **(A)** or GAPDH **(B)** as a calibrator. Concerning the peptide or supernatant treatment, for each time point, the reference sample chosen was the Dulbecco’s modified Eagle’s medium (DMEM) condition. Concerning the infection condition, for each condition, the reference sample chosen was the starting inoculation time. Data represents the mean value of fold change ± SD (n = 3).


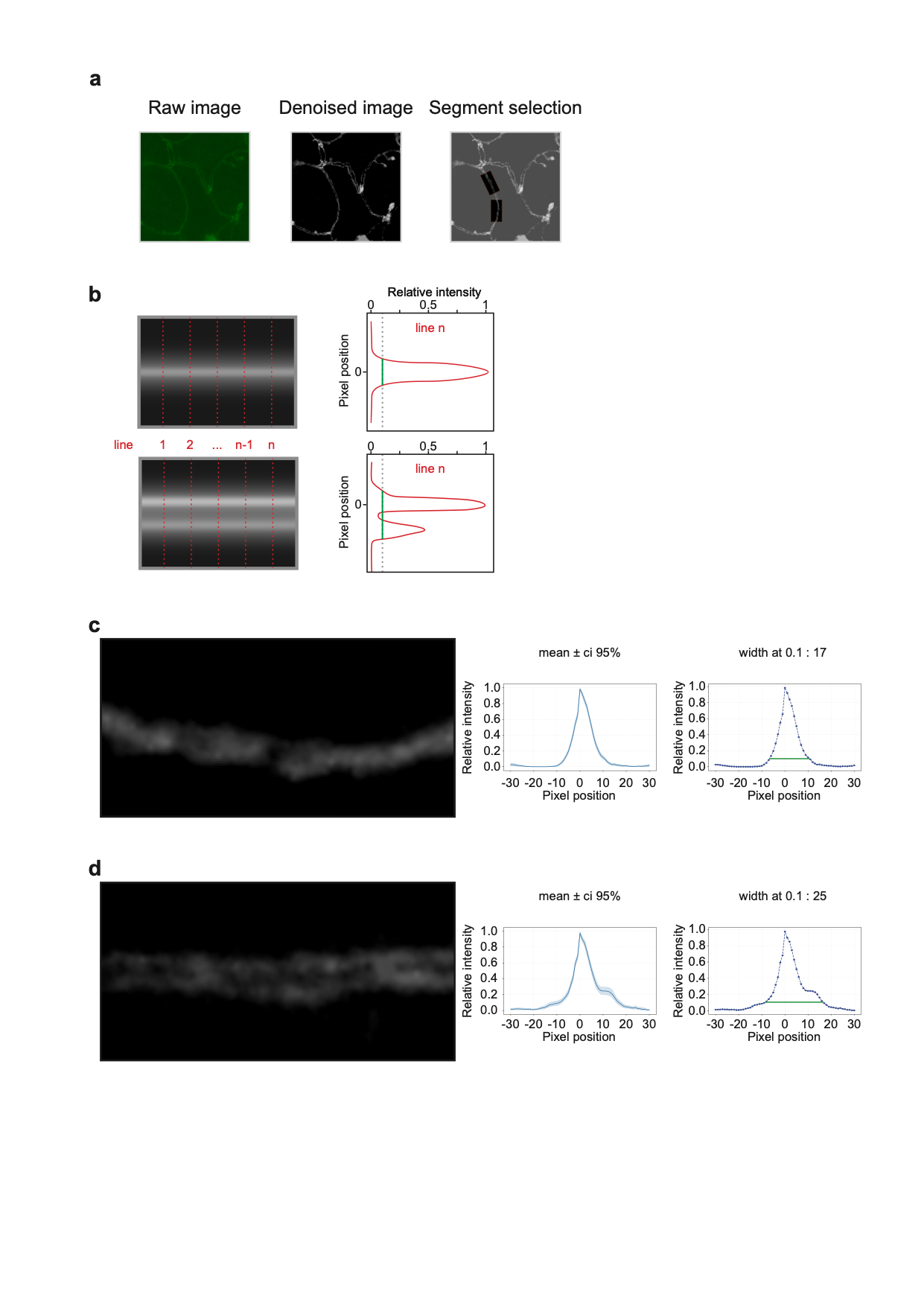
**Fig. S6.**

**Fig. S6. Details on image analysis performed on ZO-1 immuno-labelling experiments. (a)** Images depicting different steps of pre-analysis, including denoising of the raw image and the selection of region of interest. Attention was given to select segments of cell-cell contact zones. **(b)** Principle of the image analysis of ZO-1 signal. The image is chunked in small parts (red dashed lines on the theoretical images) and the signal is analyzed along a line perpendicular to the cell-cell contact zone. The shape of the signal (red solid line on the right panel) may correspond to (i) a Gaussian-like (right upper panel) corresponding to a non-disrupted ZO-1 signal, (ii) Gaussian curve with shoulder corresponding to a moderately disrupted ZO-1 signal, or (iii) a curve with multiple pics (right bottom panel) corresponding to a disrupted ZO-1 signal. For each signal, the maximum value of intensity is identified and is used to normalize the signal (intensity max = 1 and pixel position of the major pic = 0). To determine the ZO-1 signal dispersion value, the width of the curve at 10 % of the maximum intensity is determined (green solid line). **(c) & (d)** example of this strategy on two different images (left panels), corresponding to non-disturbed ZO-1 signal (c), and a disrupted ZO-1 signal (d). For each case, the mean and the 95% confidence interval was traced (central panel), and the ZO-1 signal dispersion value through the width of the curve at 10 % of the maximum intensity was determined (green solid line, right panel). In those examples, we determined a ZO-1 signal dispersion value of 17 for (c), and a ZO-1 signal dispersion value of 25 for (d).

**Fig. S7.**


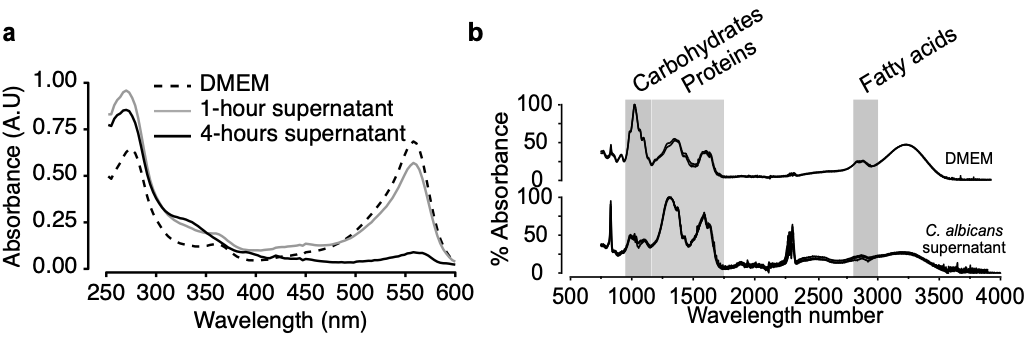


Fig. S7. Spectral absorbance of *C. albicans* supernatants. (a) Spectral absorbance of *C. albicans* supernatants obtained after 1 or 4 hours of culture were compared to DMEM. The absorbance wavelength varied from 250 to 600 nm. (b) FTIR spectra (n = 3) of DMEM medium and *C. albicans* supernatant (10^7^ yeast.mL^-1^, 4 h production). Wavelength range characteristics for carbohydrates, proteins and lipids are highlighted in grey.


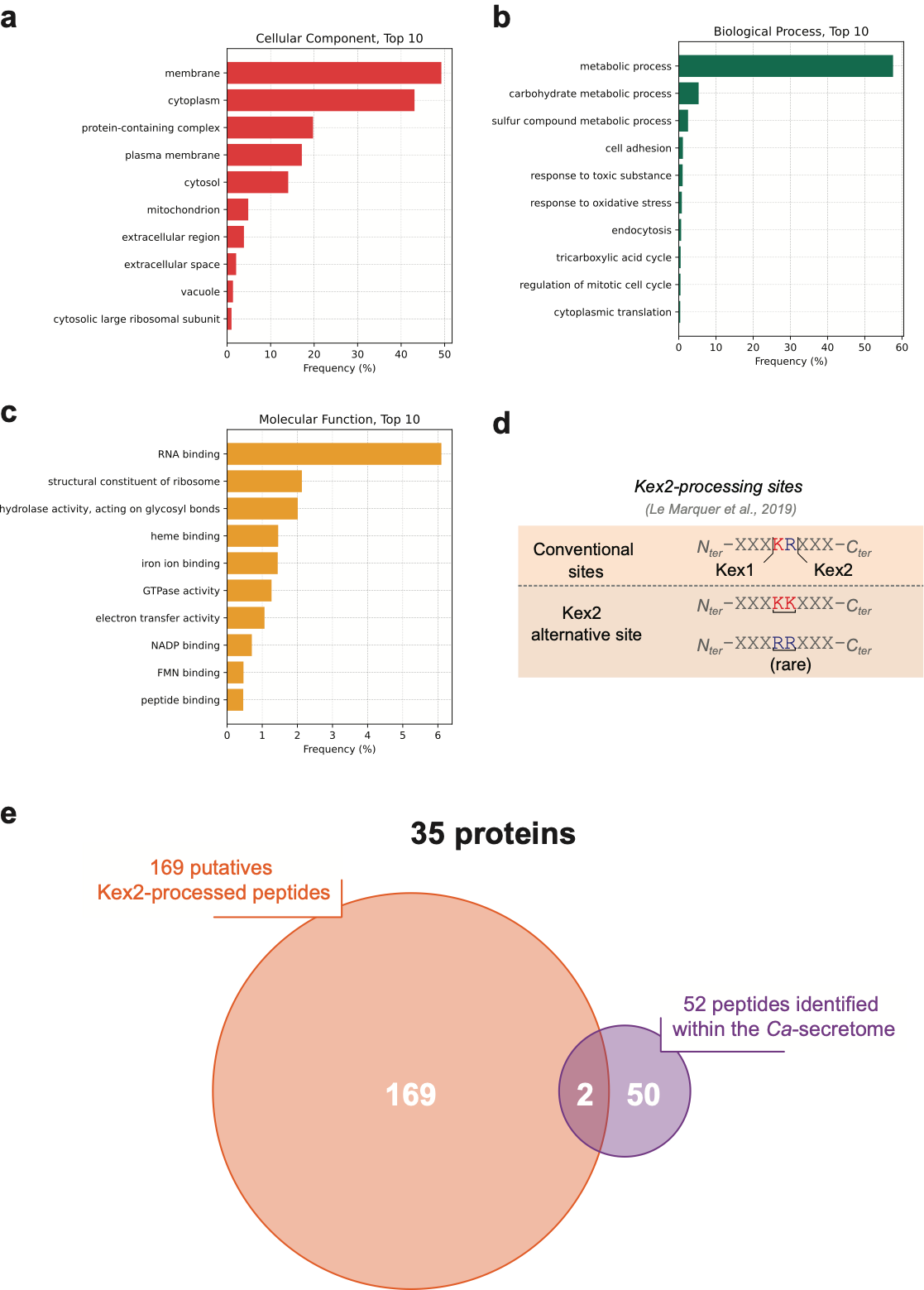
**Fig. S8.**

Fig. S8. *In silico* analysis of *C. albicans* secretome. This analysis compiles the results of three different *C. albicans* secretomes (concentrated 3-10 kDa fraction, see methods SI) and whose modulating effect on the permeability of the IEC monolayer was confirmed. Peptides of interest are the ones that were identified in all biological replicates. In total, 52 peptides were selected, corresponding to 35 proteins. GO Term analysis was performed on those proteins. The top 10 GO Terms are displayed, corresponding to the cellular component (a), the biological process (b), and molecular function (c). *In silico* cleavage of those proteins was performed, following the strategy previously described by Le Marquer *et al.* (Le Marquer *et al*. 2019) (d). Venn diagram of putative Kex2 processing peptides and peptides identified within the Ca-secretome (e).

**Fig. S9.**


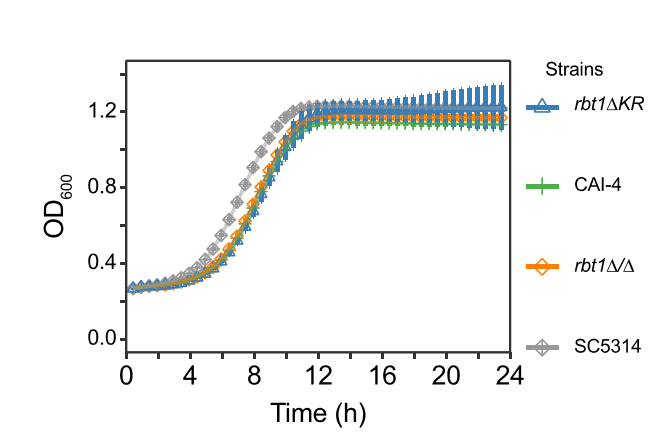

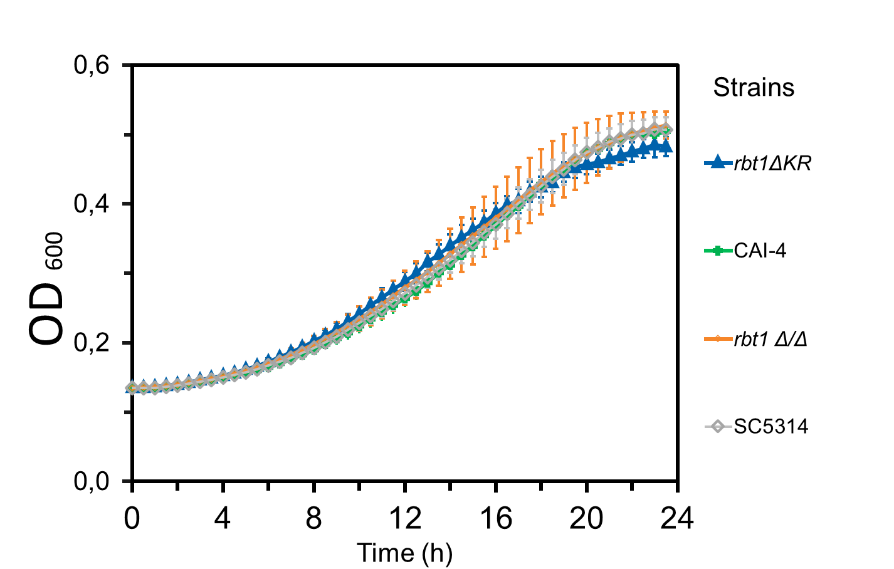


**a b**

Fig. S9. Growth curves of *C. albicans* strains and mutants. All strains were cultured in liquid DMEM w/o FBS (a) or in YPD (b) at 37°C with shaking at 300 rpm for 14 - 16 h. Stationary phase from pre-cultures were washed 3 times in PBS, and OD were adjusted at 0.1 in culture medium. A volume of 100 µL of cell suspension was placed in a 96-well plate, and growth was monitored by measuring the absorbance at 600 nm every 30 min for 100 cycles at 37°C in a microplate reader (Plate Reader infinite M200 PRO, Tecan) with orbital shaking (30 s, amplitude: 6 mm, wait: 10 s) before each measurement and multiple reads per well. At least three biological replicates were done for all experiments.

**Fig. S10.**

**
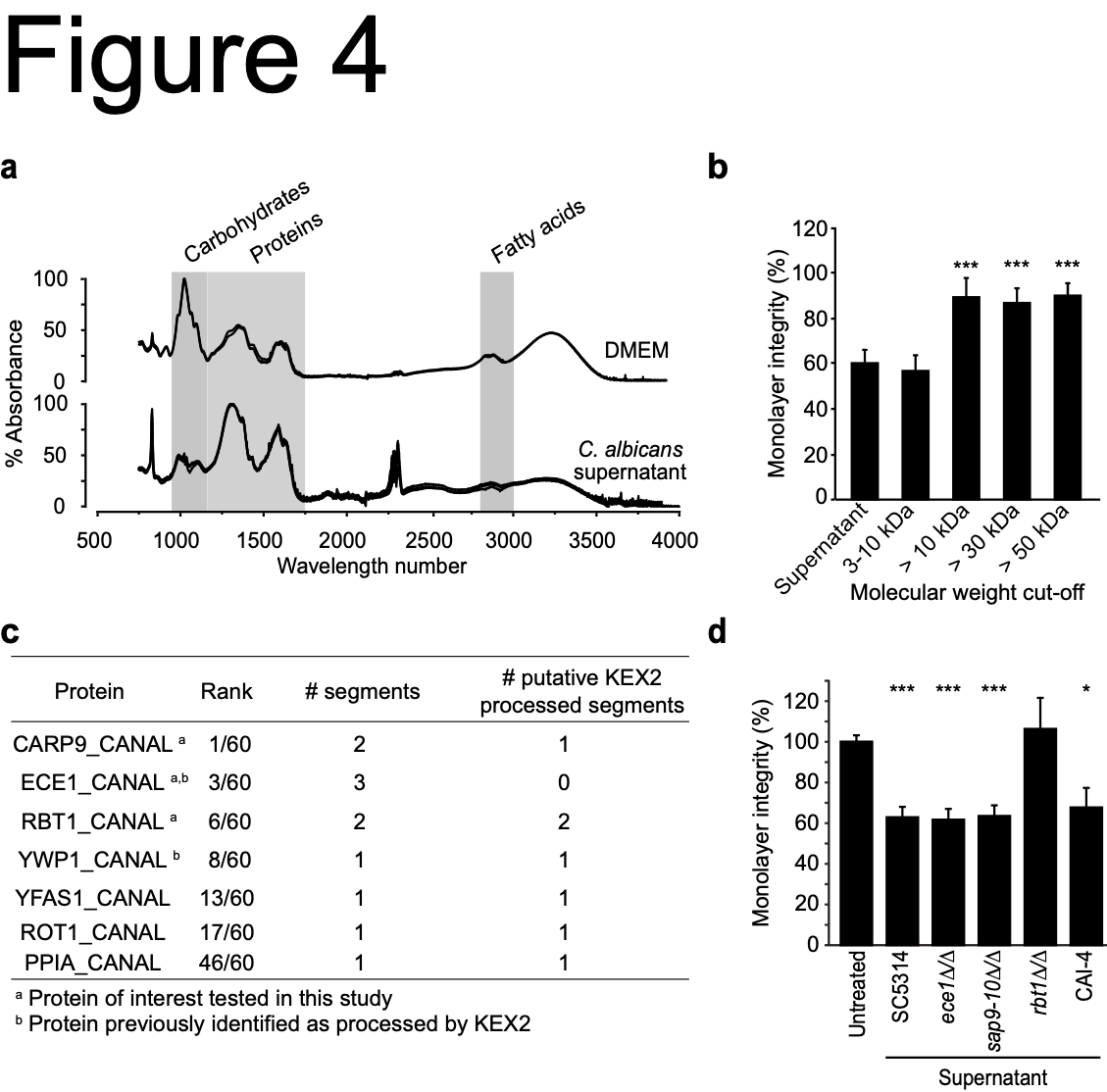
**

**Fig. S10. Identification of the putative protein of interest as modulator of monolayer integrity through TEER measurement.**

Supernatants from mutants or from parental strains were tested on differentiated Caco-2 monolayers, seeded on Transwell inserts. Cells were treated with supernatants at the apical chamber for 1 h 30. Results are displayed as the mean ± SEM percentage of variation compared to the untreated condition, and statistical analysis are performed comparing supernatants to the control condition (Dunn test,* p < 0.05, *** p < 0.001, n = 3).

**Fig. S11.**

**Fig. S11. Quantification analysis of ZO-1 protein band density from western blot.** Caco-2 monolayer was treated with *rbt1∆/∆* mutant supernatant (SN- *rbt1∆/∆*) (10^7^ yeasts.mL-1, 4h-culture) for 2, 4 or 6 hours. The membrane proteins were extracted, and 15 µg has been deposited. Results depicted here are representative of 1 experiment.

**Fig. S12.**


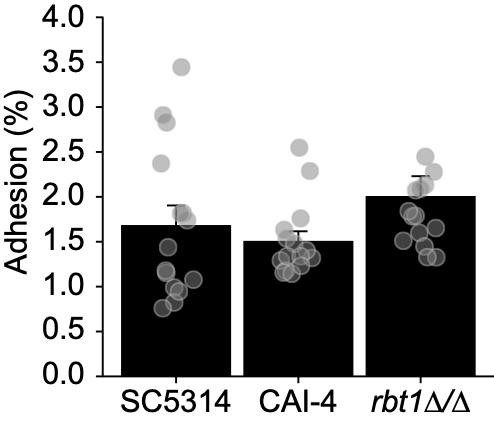


Fig. S12. *RBT1* gene deletion does not modify the adhesion property of *C. albicans* to intestinal epithelial cells. Differentiated Caco-2 cells were infected with the reference strain SC5314, the *rbt1Δ/Δ* mutant strain and its parental strain CAI-4 at MOI 0.1. Adhesion of fungal cells to Caco-2 cells was measured after 30 minutes. Results are represented as bar plots with mean value of adhesion ± SEM, and dots represent the adhesion value for each individual experiment. No statistical differences were found (Dunn test, n > 10).

Table S1.

Table S1. List of peptides identified by MS analysis within Ca-secretome, and proteins associated to them. Three different *C. albicans* secretomes (concentrated 3-10 kDa fraction, see methods SI) and whose modulating effect on the permeability of the IEC monolayer was confirmed. Peptides of interest are the ones that have been identified in all biological replicates.

| ***Sequence*** | ***Position***  ***(Start - End)*** | | ***N° accession*** | ***Protein name*** | ***Gene name*** |
| --- | --- | --- | --- | --- | --- |
| PASREDSVYLA | 2 | 12 | 1433_CANAL | 14-3-3 protein homolog | BMH1 |
| PNTLDQSYVEK | 2 | 12 | A0A1D8PCP4_CANAL | 2,4-dienoyl-CoA reductase [(3E)-enoyl-CoA-producing] | CAALFM_C102270CA |
| DVGADGQPTEELK | 117 | 129 | A0A1D8PFM0_CANAL | DUF4149 domain-containing protein | CAALFM_C113190WA |
| DTQNNDEKEEFIGVV | 661 | 675 | A0A1D8PGF0_CANAL | Lysophospholipase | PLB4.5 |
| YQQENEITPADNID | 25 | 38 | A0A1D8PGF8_CANAL | Msb2p | MSB2 |
| MDEVVPR | 1 | 7 | A0A1D8PGY5_CANAL | Probable metalloreductase AIM14 | CAALFM_C203530WA |
| DKQSDNENDAEIEQEIE | 182 | 198 | A0A1D8PIH3_CANAL | Protein BIG1 | ABG1 |
| DKQSDNENDAEIEQEIER | 182 | 199 | A0A1D8PIH3_CANAL | Protein BIG1 | ABG1 |
| VIDTGVNVK | 221 | 229 | A0A1D8PRH0_CANAL | Proteinase B | CAALFM_C703860WA |
| LTGEVPTDAQTK | 123 | 134 | A0A1D8PSH3_CANAL | Citrate synthase | CIT1 |
| EQGLADLR | 445 | 452 | ACH1_CANAL | Acetyl-CoA hydrolase | ACH1 |
| GDKVPPNTPSDEQSR | 232 | 246 | ACH1_CANAL | Acetyl-CoA hydrolase | ACH1 |
| ELAEQKDEDAKDEDKNEDEDDKEK | 715 | 738 | BMT4_CANAL | Beta-mannosyltransferase 4 | BMT4 |
| DDLSPEDDSNPR | 35 | 46 | CARP9_CANAL | Secreted aspartic protease 9 | SAP9 |
| DLSPEDDSNPRF | 36 | 47 | CARP9_CANAL | Secreted aspartic protease 9 | SAP9 |
| GESKDDLSPEDDSNP | 31 | 45 | CARP9_CANAL | Secreted aspartic protease 9 | SAP9 |
| GESKDDLSPEDDSNPR | 31 | 46 | CARP9_CANAL | Secreted aspartic protease 9 | SAP9 |
| GESKDDLSPEDDSNPRF | 31 | 47 | CARP9_CANAL | Secreted aspartic protease 9 | SAP9 |
| RGESKDDLSPEDDSNPR | 30 | 46 | CARP9_CANAL | Secreted aspartic protease 9 | SAP9 |
| RGESKDDLSPEDDSNPRF | 30 | 47 | CARP9_CANAL | Secreted aspartic protease 9 | SAP9 |
| TIETDEEKEASEKIHN | 137 | 152 | CARP9_CANAL | Secreted aspartic protease 9 | SAP9 |
| SAESALKDSQPV | 215 | 226 | CECE1_CANAL | Extent of cell elongation protein 1 | ECE1 |
| VATGVQQSIENA | 147 | 158 | CECE1_CANAL | Extent of cell elongation protein 1 | ECE1 |
| APAGVTTEVK | 279 | 288 | EF1A1_CANAL | Elongation factor 1-alpha 1 | TEF1 |
| APAGVTTEVKSV | 279 | 290 | EF1A1_CANAL | Elongation factor 1-alpha 1 | TEF1 |
| VIEDGEVGLTK | 515 | 525 | EF3_CANAL | Elongation factor 3 | CEF3 |
| VTQETEKDGTKKSSTF | 124 | 139 | HSP21_CANAL | Small heat shock protein 21 | HSP21 |
| DGRIVTGVNPQ | 210 | 220 | HSP31_CANAL | Glyoxalase 3 | GLX3 |
| NDPKTGEPLIK | 146 | 156 | HSP31_CANAL | Glyoxalase 3 | GLX3 |
| NDPKTGEPLIKGK | 146 | 158 | HSP31_CANAL | Glyoxalase 3 | GLX3 |
| FDDPEVINDAK | 76 | 86 | HSP71_CANAL | Heat shock protein HSP70 | HSP70 |
| TVPQVDGQGETEEALIQ | 21 | 37 | HWP1_CANAL | Hyphal wall protein 1 | HWP1 |
| SLHPNDPESQTEVIE | 190 | 204 | PDC1_CANAL | Pyruvate decarboxylase | PDC11 |
| GTNAPLNEEVSVH | 63 | 75 | PGA52_CANAL | Probable circularly permuted 1,3-beta-glucanase PGA52 | PGA52 |
| NAPLNEEVSVH | 65 | 75 | PGA52_CANAL | Probable circularly permuted 1,3-beta-glucanase PGA52 | PGA52 |
| YAGTDGISKA | 138 | 147 | PGA52_CANAL | Probable circularly permuted 1,3-beta-glucanase PGA52 | PGA52 |
| ETLSDDVLAK | 42 | 51 | PST1_CANAL | NAD(P)H quinone oxidoreductase PST1 | PST1 |
| LGSTDKDENDGGLAT | 245 | 259 | Q59U59_CANAL | Proteinase A | APR1 |
| NIEDTSSEDLDSR | 177 | 189 | Q5A6L5_CANAL | Cysteine proteinase 1, mitochondrial | LAP3 |
| ETVDITKL | 8 | 15 | Q5A747_CANAL | Acyl-CoA desaturase | OLE1 |
| GDDLVIDGH | 63 | 71 | Q5ADM7_CANAL | Glyceraldehyde-3-phosphate dehydrogenase | TDH3 |
| VVDLLEH | 323 | 329 | Q5ADM7_CANAL | Glyceraldehyde-3-phosphate dehydrogenase | TDH3 |
| NIEEDGEVRTQ | 117 | 127 | Q5AF37_CANAL | Uncharacterized protein | CAALFM_C402340WA |
| LEDSDGNTIVDK | 87 | 98 | Q5AND4_CANAL | Rdi1p | RDI1 |
| EAEIANKDGTIE | 61 | 72 | RBT1_CANAL | Cell wall protein RBT1 | RBT1 |
| ELDEFEELSNDGVTHS | 227 | 242 | RBT1_CANAL | Cell wall protein RBT1 | RBT1 |
| NDEVLVVR | 53 | 60 | RL26B_CANAL | Large ribosomal subunit protein uL24 | RPL26B |
| AFEADNKNI | 20 | 28 | SUN41_CANAL | Secreted beta-glucosidase SUN41 | SUN41 |
| IEEEGVALR | 116 | 124 | TSA1B_CANAL | Peroxiredoxin TSA1-B | TSA1B |
| GKTTKDDTISASVDAK | 45 | 60 | VDAC_CANAL | Mitochondrial outer membrane protein porin | POR1 |
| KNVIDPASLKEGSAEEEQKD | 72 | 91 | YFAS1_CANAL | FAS1 domain-containing protein CaO19.3004 | CAALFM_C103120WA |
| RLMGETPI | 123 | 130 | YWP1_CANAL | Yeast-form wall Protein 1 | YWP1 |
